# UPP2: fast and accurate alignment of datasets with fragmentary sequences

**DOI:** 10.1093/bioinformatics/btad007

**Published:** 2023-01-10

**Authors:** Minhyuk Park, Stefan Ivanovic, Gillian Chu, Chengze Shen, Tandy Warnow

**Affiliations:** Department of Computer Science, University of Illinois Urbana-Champaign, Urbana, IL 61820, USA; Department of Computer Science, University of Illinois Urbana-Champaign, Urbana, IL 61820, USA; Department of Computer Science, University of Illinois Urbana-Champaign, Urbana, IL 61820, USA; Department of Computer Science, University of Illinois Urbana-Champaign, Urbana, IL 61820, USA; Department of Computer Science, University of Illinois Urbana-Champaign, Urbana, IL 61820, USA

## Abstract

**Motivation:**

Multiple sequence alignment (MSA) is a basic step in many bioinformatics pipelines. However, achieving highly accurate alignments on large datasets, especially those with sequence length heterogeneity, is a challenging task. Ultra-large multiple sequence alignment using Phylogeny-aware Profiles (UPP) is a method for MSA estimation that builds an ensemble of Hidden Markov Models (eHMM) to represent an estimated alignment on the full-length sequences in the input, and then adds the remaining sequences into the alignment using selected HMMs in the ensemble. Although UPP provides good accuracy, it is computationally intensive on large datasets.

**Results:**

We present UPP2, a direct improvement on UPP. The main advance is a fast technique for selecting HMMs in the ensemble that allows us to achieve the same accuracy as UPP but with greatly reduced runtime. We show that UPP2 produces more accurate alignments compared to leading MSA methods on datasets exhibiting substantial sequence length heterogeneity and is among the most accurate otherwise.

**Availability and implementation:**

https://github.com/gillichu/sepp.

**Supplementary information:**

Supplementary data are available at *Bioinformatics* online.

## 1 Introduction

Multiple sequence alignment (MSA) is a fundamental bioinformatics task, and producing accurate alignments can have profound impact in many downstream analyses, such as phylogeny inference (Morrison and Ellis, 1997), detection of adaptive evolution (Blackburne and Whelan, 2013), or protein structure and function inference (Bork and Koonin, 1998; Ju *et al.*, 2021).

**Fig. 1.**
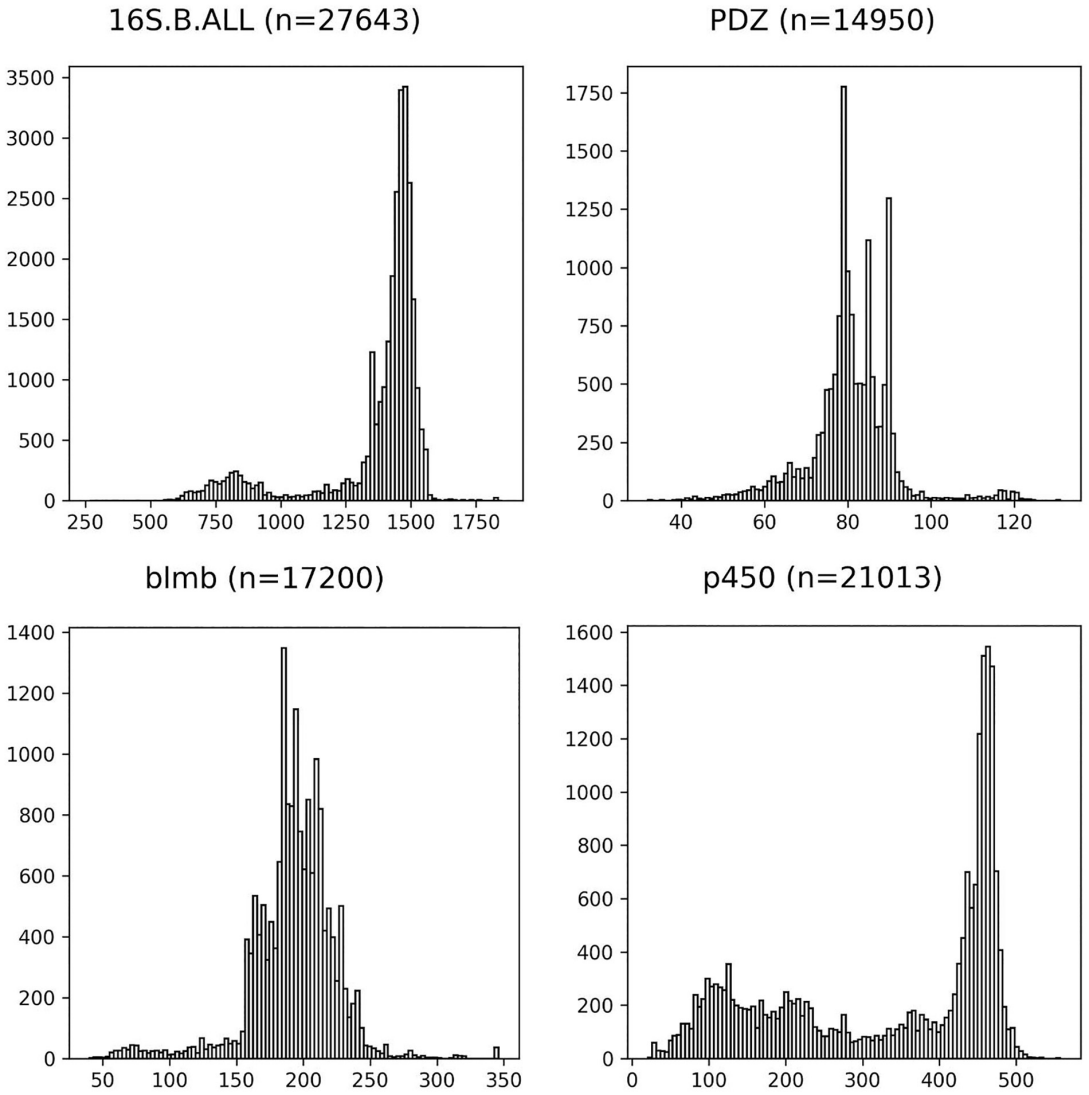
Histograms of sequence lengths in biological datasets 16S.B.ALL is from the CRW (Cannone *et al.*, 2002) and the datasets in the other three panels are from the Homfam collection (Blackshields *et al.*, 2010), which consists of HOMSTRAD reference sequences with Pfam sequences from the same domain

Because of the significant interest in alignment estimation, many alignment methods have been developed [e.g. MUSCLE (Edgar, 2004), PRANK (Löytynoja and Goldman, 2005), BAli-Phy (Suchard and Redelings, 2006), Clustal Omega (Sievers *et al.*, 2011), MAFFT (Katoh and Standley, 2013), PASTA (Mirarab *et al.*, 2015), MAGUS (Smirnov and Warnow, 2021a) and regressive T-COFFEE (Garriga *et al.*, 2019)]. However, accurate alignment is still challenging under some conditions. For example, large datasets (with many thousands of sequences) can be difficult to align with high accuracy and also present substantial computational challenges (Mirarab *et al.*, 2015; Nguyen *et al.*, 2015; Smirnov, 2021). The difficulty in aligning datasets that are highly heterogeneous due to high rates of evolution has also been documented (Liu *et al.*, 2009), but several methods (largely employing divide-and-conquer) have been able to achieve good accuracy in such conditions [e.g. PASTA (Mirarab *et al.*, 2015) and MAGUS (Smirnov and Warnow, 2021a)]. Sequence length heterogeneity (Fig. 1) introduces another challenge for alignment estimation, and one that is relatively less studied (Nguyen *et al.*, 2015; Shen *et al.*, 2021).

Ultra-large alignments using Phylogeny-aware Profiles (UPP) (Nguyen *et al.*, 2015) is a MSA method that was specifically designed to provide good accuracy on datasets with substantial sequence length heterogeneity, while maintaining scalability on large datasets. UPP operates in three basic stages: first, it extracts and aligns a subset of the sequences it deems to be full-length; second, it builds an ensemble of Hidden Markov Models (HMMs) (Durbin *et al.*, 1998) on the alignment of the selected full-length sequences; and third, it uses the ensemble to align the remaining sequences. Thus, UPP uses a combination of global MSA methods (to align the backbone sequences, which are full-length) and local MSA methods (to add the remaining sequences into the backbone alignment, which include sequences that are short).

This third step is often the bottleneck in terms of runtime. Specifically, for each additional sequence that needs to be aligned, the HMM with the highest bit-score is selected from the ensemble and is used to add the sequence into the alignment. By design, the first two steps are reasonably fast, but the third step requires an all-against-all comparison of the remaining sequences against the HMMs in the ensemble. Thus, the runtime of UPP can be prohibitively high when there are many sequences that are not full-length and when the ensemble contains many HMMs.

In the last year, modifications to UPP to improve its accuracy and theoretical foundation have been explored. The default for UPP as provided in the github site uses PASTA to align the backbone sequences. However, Shen *et al.* (2021) showed that alignment accuracy was improved by using MAGUS instead of PASTA to compute the backbone alignment. Another potential weakness in the original UPP approach is the use of the bit-score to select the single HMM to align the query sequence. A bit-score represents the log likelihood ratio of a query sequence being emitted by an HMM to the likelihood of a query sequence being emitted by a null HMM. However, the bit-score does not correspond to the probability that the query sequence is generated by the selected HMM from the ensemble, as this specific question depends also on the number of sequences used to build the HMM as well as the ensemble of HMMs that has been constructed. To address this, a modification to the use of bit-scores, called ‘adjusted bit-scores’, was presented in Shen *et al.* (2022). Under the assumption that exactly one of the HMMs in the ensemble generated the query sequence, adjusted bit-scores can be interpreted as probabilities that the given HMM generates the query sequence (Shen *et al.*, 2022). Supplementary Section S2 provides the formula for the adjusted bit-score, its derivation and additional discussion.

Although the version in the UPP Github site still uses PASTA for the backbone and selects the best HMM based on raw bit-scores, based on these two studies, the current recommended setting for UPP uses MAGUS for the backbone alignment and selects the ‘best’ HMM from the ensemble based on the adjusted bit-score.

These modifications aimed to improve accuracy rather than runtime, and UPP has remained computationally intensive as a result of its all-against-all algorithmic design. Here, we present UPP2, a modification to UPP that is designed to reduce its runtime and improve its scalability to large sequence datasets. The main modification, we use is a replacement of the all-against-all comparison of query sequences and HMMs by a much smaller number of comparisons, so that each query sequence is scored against a logarithmic number of HMMs instead of against all the HMMs. As we will show, this change reduces the runtime, sometimes dramatically, without hurting accuracy.

## 2 UPP2

### 2.1 The UPP three-stage pipeline

In the first stage, it computes a backbone alignment (using PASTA or MAGUS) and backbone tree [using FastTree (Price *et al.*, 2010)] on a subset of the input sequences, in the second stage, it builds an ensemble of profile HMMs on the backbone alignment and in the third stage, it uses the ensemble to add all the remaining sequences into the backbone alignment using commands from HMMER (Eddy, 2011) (hmmbuild, hmmsearch and hmmalign). Here, we provide some additional details.

For Stage 1, by default, UPP will select up to 1000 sequences to include in its backbone, and these sequences are selected at random from the set of sequences within 25% in length of the median length sequence. The alignment is built using a selected ‘base method’, with PASTA the original technique and now MAGUS the recommended technique. Our own studies have suggested that larger backbones may improve final alignment accuracy; hence, using 10 000 sequences for the backbone on large datasets (e.g. with at least 25 000 sequences) is the approach that we follow in this study.

For Stage 2, UPP computes a set of subset alignments by hierarchically decomposing the backbone tree at a centroid edge (i.e. an edge that splits the leaf set into two sets of roughly equal sizes) until all the subtrees are at most size *z*, where *z* is an input to UPP. UPP builds an HMM on each set created during this decomposition, including the full set, thus producing a collection of HMMs that we refer to as the ‘ensemble of HMMs’ (eHMM) for the backbone alignment. In the initial version of UPP, *z* was set to 10. Some studies (Mirarab *et al.*, 2012) that developed eHMMs for other purposes have suggested that smaller values (e.g. z=2) might improve accuracy, but a more recent study exploring this question for alignment estimation (Shen *et al.*, 2021) has found otherwise.

For Stage 3, UPP adds every additional sequence (i.e. ones that are not in the backbone) into the backbone alignment. These additional sequences are referred to as ‘query sequences’ and are added as follows. For each query sequence, *hmmsearch* is used to find the HMM that returns the highest bit-score (the original setting) or the highest adjusted bit-score (the current recommendation). Then, each query sequence is added into the subset alignment used to construct the selected HMM using *hmmalign*. Since the subset alignments are induced by the backbone alignment, this also means the query sequence can be added into the backbone alignment as well. The addition of the query sequence into the backbone alignment defines an ‘extended alignment’. The extended alignments from the different query sequences are merged together using transitivity, thus producing a final alignment containing all the sequences.

### 2.2 UPP2: modifying Stage 3 to improve speed

**Fig. 2.**
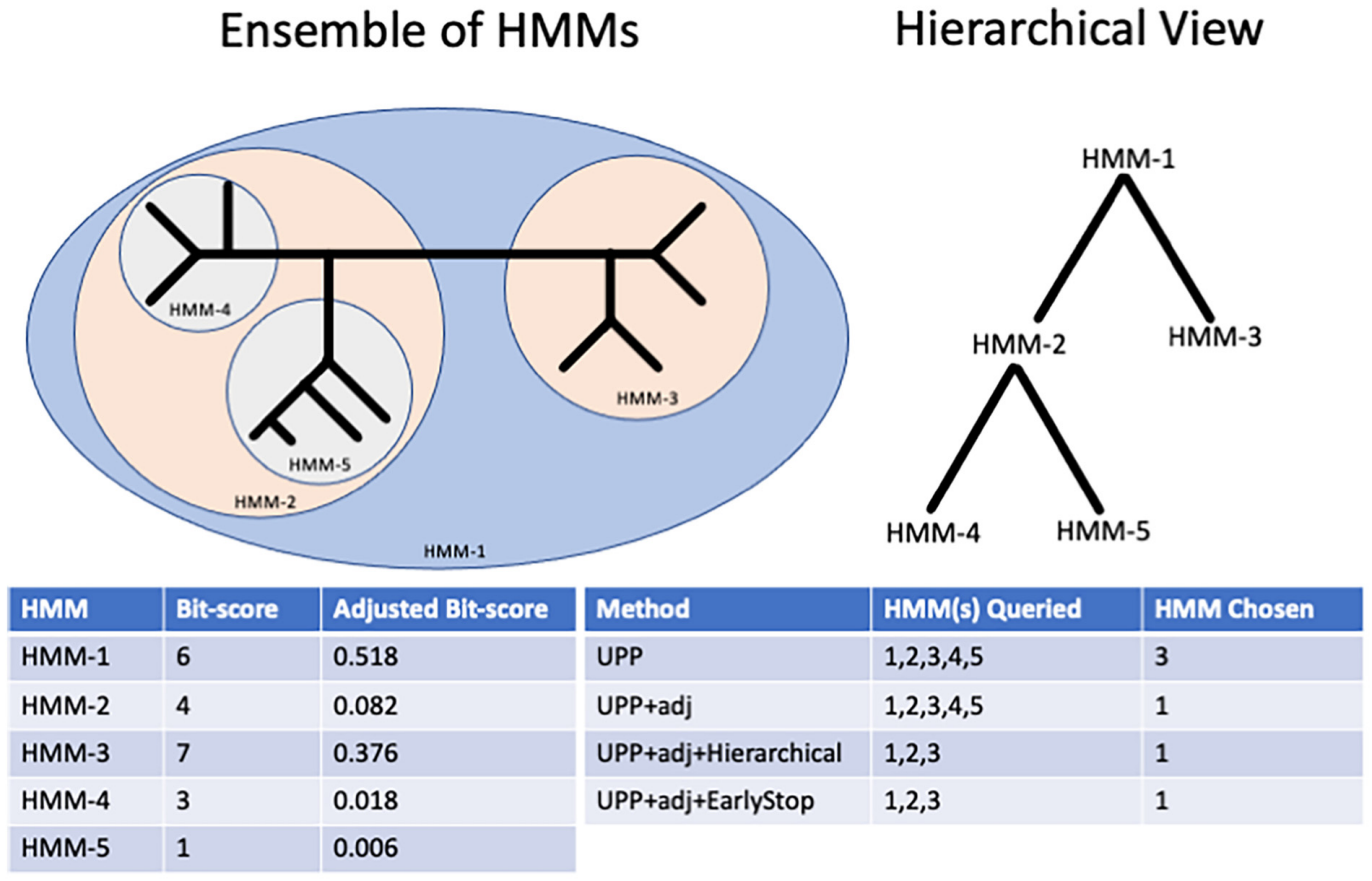
UPP and UPP2 search strategies (toy example). Here, we show a sample ensemble of HMMs and how different search strategies pick different HMMs within the ensemble. UPP and UPP+adj by default search through every HMM but use different criteria (raw bit-scores or adjusted bit-scores, respectively). UPP will choose HMM-3 since HMM-3 has the highest bit-score while UPP+adj will choose HMM-1 since HMM-1 has the highest adjusted bit-score. UPP+adj+Hierarchical will start at HMM-1 and descend down the subtree with the highest adjusted bit-score. UPP+adj+EarlyStop will descend down the subtree with the highest adjusted bit-score and stop once all immediate children HMMs have worse adjusted bit-scores than the current best HMM

In Stage 3, each query sequence picks a best HMM (based on the bit-score or the adjusted bit-score) and then that HMM is used to add the query sequence into the backbone alignment. Due to its hierarchical decomposition strategy, UPP produces many HMMs, all of which have to be compared against every single query sequence. This quickly presents scalability issues in several cases: as the size of backbone increases, as *z* (which defines the decomposition stopping rule) decreases, or as the number of query sequences increases. We propose two strategies (‘Hierarchical’ and ‘EarlyStop’) based on this hierarchical decomposition strategy to speed up the search: Hierarchical and EarlyStop (Fig. 2). We denote UPP with these strategies using the notation ‘UPP+Hierarchical’ or ‘UPP+EarlyStop’.

*Hierarchical:* Stage 2 defines a hierarchy of HMMs based on their sequence sets, so that the set of HMMs forms a rooted tree. Here, we describe how the Hierarchical Search strategy operates, using adjusted bit-scores. To select an HMM for a given query sequence *q*, we start at the root HMM and we compute its adjusted bit-score given *q*. We then evaluate the children HMMs and descend down into the subtree that has the larger adjusted bit-score (randomly selecting one in the case of a tie). The process continues until a leaf HMM is reached. The HMM with the largest adjusted bit-score (i.e. the HMM deemed the most likely to have emitted the query sequence) encountered during the traversal then becomes the selected HMM for the query sequence. Note that this strategy evaluates at most two HMMs per level in the tree. In the case of a tie, the HMM that comes first in a pre-order traversal is chosen.

*EarlyStop:* We follow the same basic strategy as Hierarchical. However, the process stops descending down the subtree in the hierarchical search process if both of the two children HMMs have lower adjusted bit-scores, and are therefore considered less likely to have emitted the query sequence than the current best HMM (hence the name ‘EarlyStop’).

## 3 Experimental study

**Overview.** We performed two experiments, one for designing UPP2 (Experiment 1) and one for evaluating UPP2 in comparison to leading alignment methods (Experiment 2). Experiment 1 was performed on a small set of ‘training datasets’ and Experiment 2 was performed on a larger set of ‘testing datasets’. Methods were evaluated for alignment error and runtime.

**Alignment methods.** We evaluated variants of UPP2 that differ in terms of the backbone alignment method (PASTA or MAGUS), the use of raw or adjusted bit-scores, the stopping condition (i.e. how *z* is set), and whether the all-against-all comparisons are performed or one of the two faster search strategies is used. Recall that the original version of UPP uses PASTA backbones, raw bit-scores, sets z=10, and performs all-against-all comparisons to find the best HMM for each query sequence. We explore the following variants of UPP2 (indicating how they differ from the original version of UPP below). Each of these versions can have either PASTA or MAGUS backbones, as indicated in parentheses.

- *UPP+adj*: UPP+adj differs from the original UPP by using adjusted bit-scores.
- *UPP+adj+Hierarchical*: identical to UPP+adj except that it uses the Hierarchical search strategy.
- *UPP+adj+EarlyStop*: identical to UPP+adj except that it uses the EarlyStop search strategy.

We also evaluated the following alignment methods (see Supplementary Section S3 for commands):

- MUSCLE (3.8.31), limited to two iterations.
- Clustal Omega (1.2.4), used in its default mode.
- T-COFFEE (13.45.0.4846264), used in the default regressive mode.
- MAGUS (commit on 4/5/21, commit ID in the Supplementary Material) used in its default mode, which is with recursion in the newest version of MAGUS (Smirnov, 2021; Smirnov and Warnow, 2021a).
- PASTA (v1.9.0), used in its default mode.
- MAFFT (7.487), with the *linsi* mode used for small to medium datasets and *auto* mode used for the largest datasets; we also included xinsi, qinsi and ginsi on the RNA datasets.
- UPP (through appropriate settings of the algorithmic parameters within the UPP2 code).

**Datasets.** We used both biological and simulated datasets (both nucleotides and proteins) for the experiments, separating them into the training datasets (used in Experiment 1) and the testing datasets (used in Experiment 2). We had fragmentary versions of the datasets, where the suffix ‘HF’ denotes high fragmentary datasets; these are constructed by taking the original dataset and making half of the sequences ∼25% of the original median sequence length. The fragmentation process is explained in full detail in Smirnov and Warnow (2021b). The empirical statistics (i.e. number of sequences, average sequence length, percent of the reference alignment occupied by gaps, and average and maximum *p*-distance) for these datasets are provided in Supplementary Tables S1 (for the simulated and nucleotide datasets) and S2 (for the protein datasets). The sequence length histograms for the biological datasets are provided in Supplementary Figures S1–S20.

The ROSE simulated datasets, introduced in Liu *et al.* (2009), are 1000-sequence datasets with varying gap lengths, which are denoted by ‘S’ for short gap lengths, ‘M’ for medium gap lengths and ‘L’ for long gap lengths. We used 1000S1 through 1000S5, 1000M1 through 1000M5 and 1000L1 through 1000L5 as well as their high fragmentary counterparts 1000S1-HF through 1000S5-HF, 1000M1-HF through 1000M5-HF and 1000L1-HF through 1000L5-HF. 1000M1 and 1000M1-HF were using for training while the other model conditions were reserved for testing.

The RNASim datasets are created by sampling from the RNASim million-sequence dataset, originally created by Guo *et al.* (2009) and studied in Mirarab *et al.* (2015). The RNASim sequences evolve under a substitution and indel model that includes selection to maintain secondary structures. We used RNASim1000 and RNASim1000-HF, the same datasets as used in Smirnov and Warnow (2021b), which are published at https://doi.org/10.5061/dryad.95x69p8h8.

We include 10 RNA datasets from the Comparative Ribosomal Website (CRW) (Cannone *et al.*, 2002), which have reference alignments based on secondary structure. These datasets vary in size and are based on the 16S, 23S and 5S genes. The three largest of these datasets, 16S.B.ALL, 16S.3, and 16S.T, were used in Liu *et al.* (2012) to evaluate methods for large-scale alignment and are available with reference alignments at https://sites.google.com/eng.ucsd.edu/datasets/alignment/16s23s. The remaining datasets and reference alignments (i.e. 16S.A, 16S.C, 16S.M, 23S.A, 23S.C, 23S.M and 5S.3) are available at https://crw-site.chemistry.gatech.edu/DAT/3C/Alignment/.

We include 10 protein datasets from the Homfam collection (Blackshields *et al.*, 2010). These datasets were created by combining small numbers of HOMSTRAD reference sequences with Pfam sequences from the same domain, so that their reference alignments are only on the HOMSTRAD sequences. These were used in the study by Mirarab *et al.* (2015) and are available at https://sites.google.com/eng.ucsd.edu/datasets/alignment/pastaupp.

For the training datasets (Experiment 1), we used two model conditions, 1000M1 (one of the model conditions from the ROSE simulated datasets with high rates of evolution) and RNASim1000. For each model condition, we explored full-length versions and HF versions. We used the remaining datasets as the testing datasets (Experiment 2).

**Alignment error.** We used FastSP (1.7.1) (Mirarab and Warnow, 2011) for calculating SPFN and SPFP error rates of estimated alignments relative to the reference alignments, defined as follows. SPFN refers to ‘sum-of-pairs false negatives’, and is the number of the pairwise homologies found in the reference alignment but not in the estimated alignment, while SPFP refers to ‘sum-of-pairs false positives’ and is the number of pairwise homologies found in the estimated alignment but not in the reference alignment. These are normalized by the number of homologies in the reference alignment or estimated alignment, respectively, to produce the SPFN and SPFP error rates.

**Experiment 1: Overview.** Experiment 1 explored the design space of UPP2 (decomposition size, use of bitscores or adjusted bitscores, use of MAGUS or PASTA for the backbone alignment, and choice of search strategy), using the testing datasets. We used Experiment 1 to specify to set the algorithmic parameters for the approach and refer to this variant as ‘UPP2’.

**Experiment 2: Overview.** Experiment 2 compared UPP2, UPP(MAGUS)+adj, MAGUS, PASTA, MAFFT (using *linsi* for datasets with at most 1000 sequences and *auto* otherwise), Clustal Omega, regressive T-COFFEE, and MUSCLE using the testing datasets (both biological and simulated). Experiment 2a examined results on simulated datasets with fragmentary sequences, Experiment 2b examined results on 10 RNA datasets from the CRW and Experiment 2c examined results on 10 Homfam datasets. Because some biological datasets exhibit severe sequence length heterogeneity (e.g. 23S.A from the CRW collection, Supplementary Fig. S7), the selection of backbone sequences on the biological datasets was performed using a sliding window procedure (described in Supplementary Section S7). In brief, by varying *L*, we selected a sequence length *L* that maximized the number of sequences within 25% of *L*. The backbone sequence set then included any sequence of length at least 75% *L*. In all other regards, we followed the same procedure as for Experiment 1.

**Computational resources.** Within a given experiment, all analyses were run under either Blue Waters (Bode *et al.*, 2013) or on the Campus Cluster at UIUC. All methods were limited to a maximum of 7 days of wall-time, 16 cores and 256 GB of RAM. MUSCLE does not have a multi-threaded version and was unable to take advantage of the core count. Some failures to complete due to limitations of time and/or memory occurred; these are reported in detail in Supplementary Section S6.

## 4 Results

### 4.1 Experiment 1: designing UPP2

In this first experiment, we evaluated variants of UPP2, varying (i) use of adjusted or raw bit-scores, (ii) using MAGUS or PASTA backbones, (iii) changing the value for *z* (maximum allowed size of subsets before decomposition stops) and (iv) use of EarlyStop or Hierarchical as opposed to all-against-all. On all the datasets, we explored (i.e. full-length and also HF versions of 1000M1 and RNASim1000), there were no noteworthy differences in alignment accuracy for any of these modifications, with the exception that using MAGUS instead of PASTA for the backbone alignment improved accuracy (Supplementary Figs S21–S23).

We also saw that using MAGUS instead of PASTA for the backbone alignment reduced runtime (Supplementary Fig. S22) and that using the new search strategies (EarlyStop or Hierarchical) improved runtime even further (Supplementary Figs S23 and S24). Specifically, UPP(PASTA)+adj+Hierarchical reduced the runtime by a large margin compared to UPP(PASTA)+adj and UPP(PASTA)+adj+EarlyStop further improved runtime compared to UPP(PASTA)+adj+Hierarchical.

The runtime improvement obtained through the use of Hierarchical or EarlyStop is not surprising, but the achievement of comparable accuracy was not guaranteed. Although we did not see a difference in accuracy between z=2 compared to z=10, because previous studies [e.g. Mirarab *et al.* (2012)] have suggested the potential for this setting to improve accuracy, we set z=2 for the default for all datasets. Our final default settings for the algorithmic parameters are to use: (i) adjusted bit-scores, (ii) MAGUS for the backbone alignment and (iii) EarlyStop for the search strategy. We denote this variant simply as ‘UPP2’. We use ‘UPP(MAGUS)+adj’ to refer to UPP with adjusted bit-scores and MAGUS backbone alignments.

### 4.2 Experiment 2: UPP2 compared to benchmark methods

In this experiment, we compared UPP2 to other alignment methods on the testing datasets. Experiment 2a explored results on simulated datasets with fragmentary sequences, Experiment 2b explored results on large 16S datasets, Experiment 2c explored results on Homfam datasets, and Experiment 2d explored results on small to medium RNA datasets from the CRW (Cannone *et al.*, 2002).

**Fig. 3.**
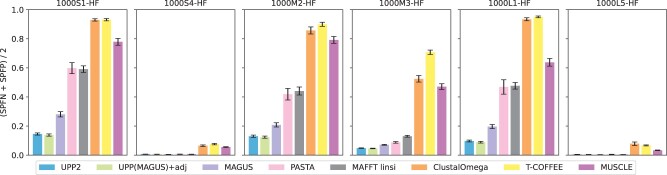
Experiment 2a: comparison of UPP2 to other MSA methods on simulated fragmentary datasets. All methods except T-COFFEE and MUSCLE were run in their default modes and with 16 threads, when possible. T-COFFEE was run using the default regressive mode and MUSCLE was limited to two iterations. UPP(MAGUS)+adj and UPP2 both use MAGUS backbone alignments, FastTree backbone trees and adjusted bit-scores, but they differ in their search strategies (EarlyStop or all-against-all). All datasets have 20 replicates each. The means are shown with error bars indicating standard error for alignment error

**Fig. 4.**
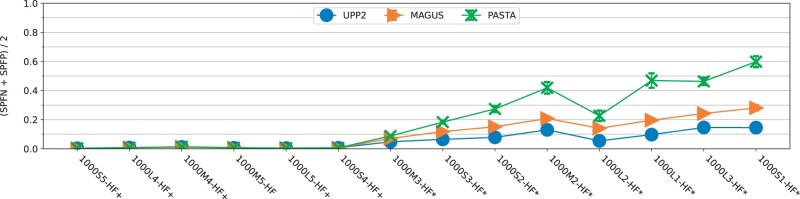
Experiment 2a: alignment error (means across 20 replicates) of UPP2, MAGUS and PASTA on simulated 1000-sequence datasets with fragmentary sequences. At α=0.05, asterisks denote the model conditions on which UPP2 was statistically significantly better than MAGUS while plus symbols denote the model conditions on which MAGUS was statistically significantly better than UPP2; *P*-values are provided in Supplementary Table S3. The error bars indicate standard error

**Experiment 2a: Results on simulated datasets with fragmentation.** In this experiment, we evaluated UPP2 to other alignment methods on the ROSE simulated datasets with fragmentary sequences; Figure 3 shows results for all methods on a representative sample of six model conditions, and Figure 4 shows results just for the three best methods on all 14 model conditions. On these datasets, UPP2 and UPP(MAGUS)+adj were the most accurate, followed by MAGUS. PASTA and MAFFT had comparable accuracy to each other and trailed behind the leading group of three methods. Clustal Omega, T-COFFEE, and MUSCLE were the least accurate methods, but MUSCLE was somewhat more accurate than the others, and Clustal Omega and T-COFFEE tended to perform similar to each other. There is a slight runtime advantage of using UPP2 over UPP(MAGUS)+adj on these datasets (Supplementary Fig. S25). Figure 4 compares the top three methods, UPP2, MAGUS and PASTA, on all 14 ROSE model conditions. We use an ordering on the model conditions from Liu *et al.* (2009) so that alignment error rates generally increase from left-to-right. While the three methods have nearly perfect alignment error and are close to identical on the six easiest model conditions (i.e. the leftmost conditions), as we move from left-to-right, we see error rates increasing for all methods. However, UPP2 error rates increase more slowly than for the others, and UPP2 has the best accuracy of these three. Thus, across all the more difficult model conditions, we see that UPP2 is much more accurate than MAGUS, which in turn is more accurate than PASTA. Furthermore, the difference in accuracy between UPP2 and the next best method is often very large. In addition, while there are conditions where MAGUS is statistically significantly more accurate than UPP2, those conditions are also ones where alignment error rates are below 0.1%.

An examination of the properties of these model conditions (Supplementary Table S1) shows that these conditions vary significantly in terms of average and maximum *p*-distances (i.e. normalized Hamming distances), and that these average *p*-distances generally increase as we move from left-to-right. Thus, increases in average *p*-distance result in increases in alignment error for all methods, and also increase the gap between methods.

**Experiment 2b: Results on RNA datasets.** In this experiment, we explored results on 10 RNA datasets from the CRW.

We first explored alignments on the seven smallest datasets. As shown in Supplementary Figure S28, MAFFT-linsi and MAFFT-ginsi were overall the most accurate of the MAFFT variants, with a small advantage to MAFFT-ginsi (Supplementary Table S6). A comparison between the other methods (Supplementary Figs S29 and S30) show UPP2, UPP(MAGUS+adj) and MAGUS having the best accuracy of all methods, with PASTA, MAFFT-linsi and MAFFT-ginsi following fairly closely behind. MUSCLE, T-Coffee and Clustal Omega are less accurate than the other methods. UPP2 tended to be slightly faster than UPP(MAGUS)+adj.

**Fig. 5.**
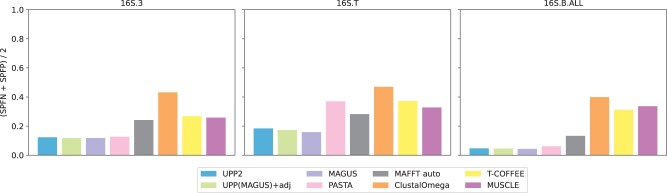
Experiment 2b: comparison of UPP2 to other MSA methods on three largest RNA datasets. The three datasets are from the CRW (Cannone *et al.*, 2002). 16S.3 has 6323 sequences, 16S.T has 7350 sequences and 16S.B.ALL has 27 643 sequences. MAFFT *auto* mode was used rather than the *linsi* mode due to the large dataset sizes

**Fig. 6.**
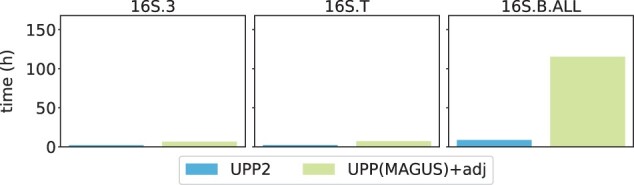
Experiment 2b: runtime comparison of UPP2 to UPP(MAGUS)+adj on three largest RNA datasets. The three datasets are from the CRW (Cannone *et al.*, 2002). One thousand sequences were chosen for the backbone for 16S.3 and 16S.T while 10 000 sequences were chosen for the backbone for 16S.B.ALL. 16S.3 has 6323 sequences, 16S.T has 7350 sequences and 16S.B.ALL has 27 643 sequences

Results on three largest of these datasets (Fig. 5) show that MAFFT-xinsi, MAFFT-qinsi and MAFFT-ginsi all failed to run due to time or memory issues. UPP2 and MAGUS were the most accurate methods, while PASTA was as accurate as the top methods on 16S.B.ALL and 16S.3 but not on 16S.T. UPP(MAGUS)+adj was as accurate as UPP2 on these datasets but took far more time compared to UPP2 (about 110 h compared to 10 h) on the 16S.B.ALL dataset. Clustal Omega had the highest alignment error across all large 16S datasets. T-COFFEE, MUSCLE and MAFFT all performed similarly to each other on 16S.3, but MAFFT was able to beat the other two methods on 16S.B.ALL and 16S.T. Although UPP(MAGUS)+adj tied for most accurate when it could complete, it was vastly slower on the largest 16S dataset. UPP2 and MAGUS reliably had good accuracy (tying for best) and completed within reasonable times. The comparison between UPP2 and MAGUS shows indistinguishable accuracy on these datasets (Fig. 6).

**Fig. 7.**
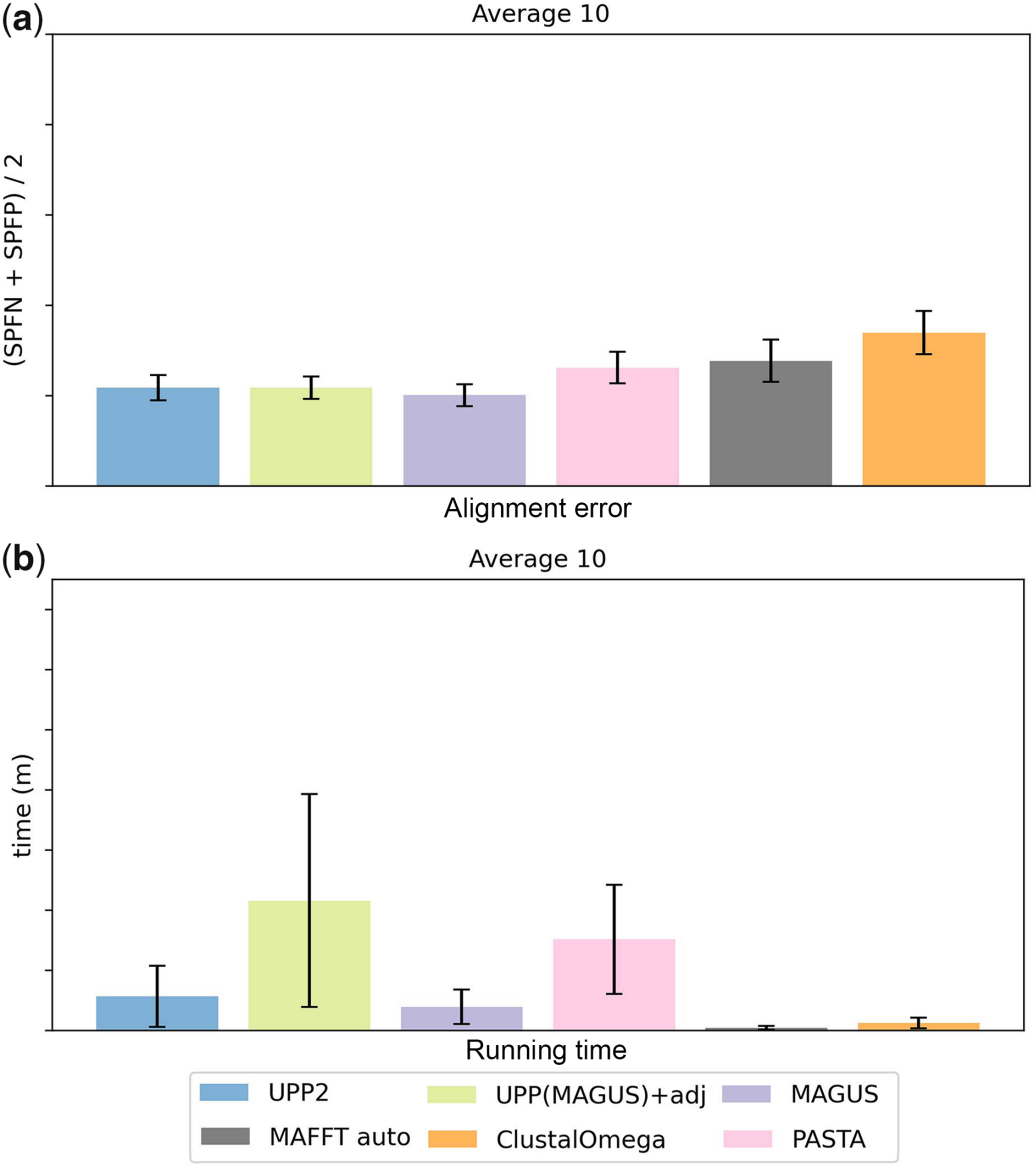
Experiment 2c: comparison of UPP2 to other MSA methods on Homfam datasets. MUSCLE could not run on the two largest datasets (zf-CCHH and rvp), but the other methods completed on all the datasets. MUSCLE had higher error on the eight datasets on which it could run than all the other methods and was also among the slowest. We show (**a**) average alignment error and (**b**) runtime for all methods on the 10 datasets with MUSCLE omitted; see Supplementary Figure S27 for results with MUSCLE. The number of sequences per dataset is as follows: PDZ (14 950), blmb (17 200), p450 (21 013), adh (21 331), aat (25 100), rrm (27 610), Acetyltransf (46 285), sdr (50 157), zf-CCHH (88 345) and rvp (93 681). MAFFT *auto* mode was used rather than the *linsi* mode due to the large dataset sizes

**Experiment 2c: Results on Homfam datasets.** We provide a comparison of average performance (alignment error and runtime) for methods, averaged across the 10 largest Homfam datasets (Fig. 7); results for individual Homfam datasets are provided in Supplementary Figure S26 and Supplementary Tables S4 and S5. MUSCLE failed to run on 2 of the 10 datasets (memory issues), and so results including MUSCLE are restricted to the 8 datasets on which it could run, and shown in Supplementary Figure S27. On those datasets, it had higher error than the other methods and was also slower. T-COFFEE using regressive in its default mode could not complete on any of the 10 datasets, with 8 failures due to memory and the other 2 failures due to other issues. See Supplementary Section S6 for full details.

The trends for the remaining methods are as follows. UPP2, UPP(MAGUS)+adj and MAGUS were the most accurate methods, with a slight advantage to MAGUS. PASTA and MAFFT–auto were close and slightly less accurate than the top three methods. Clustal Omega was less accurate than both PASTA and MAFFT–auto, and MAFFT–auto was the fastest with Clustal Omega only slightly slower, followed closely by MAGUS. UPP2 was somewhat slower and then UPP(MAGUS)+adj was the slowest.

## 5 Discussion

The major difference between UPP2 and UPP(MAGUS)+adj is the replacement of the all-against-all search strategy by EarlyStop. This difference did not seem to impact accuracy, but did allow UPP2 to have a runtime advantage over UPP(MAGUS)+adj that can be very large in some conditions. For example, on 16S.B.ALL, UPP2 is able to complete its analysis several days before UPP(MAGUS)+adj is able to complete, and has the same alignment accuracy. However, on some small datasets, the runtime advantage, although present, is reduced.

UPP2 and UPP(MAGUS)+adj tend to have the best accuracy of all tested methods on datasets with fragmentation, but MAGUS is very close (and sometimes better). However, the relative accuracy depends on the degree of fragmentation in the dataset as well as the rate of evolution (as reflected in the average *p*-distance). When there are only a small number of short sequences or when the rate of evolution is sufficiently low, then MAGUS can be as accurate as UPP2 and can even surpass UPP2 in accuracy. However, UPP2 provides an accuracy advantage over MAGUS and other standard MSA methods for those datasets exhibiting both high rates of evolution and fragmentation. The close accuracy between UPP2 and MAGUS is the result of UPP2 using MAGUS to align the backbone sequences, and is unsurprising.

## 6 Conclusions

The estimation of MSAs on large datasets is a common step in much biological discovery. However, many modern biological datasets exhibit substantial sequence length heterogeneity, and only a few methods have been able to provide good accuracy under these conditions. UPP (Nguyen *et al.*, 2015) is a well-established method for aligning datasets with short sequences, but UPP’s all-against-all approach makes it computationally intensive. By replacing this search strategy with the EarlyStop approach, UPP2 achieves the same high accuracy but is much faster than UPP(MAGUS)+adj. Thus, this study suggests that UPP2 is a useful method for MSA that generally matches or improves on the accuracy of other methods on large datasets when substantial sequence length heterogeneity is present.

Finally, this study and others have shown that some accurate methods are limited to small datasets (e.g. MAFFT-ginsi and MAFFT-lini), but can run on large datasets when incorporated within MAGUS and subsequently into UPP and UPP2. Hence, one direction for future work is to investigate the impact of replacing MAFFT-linsi by MAFFT-ginsi within MAGUS, and subsequently within UPP2. Future enhancements to UPP2 could include the use of other techniques than HMMER for building the ensemble of HMMs to represent the backbone alignment [e.g. HH-suite (Steinegger *et al.*, 2019) and Infernal (Nawrocki and Eddy, 2013)].

## Supplementary Material

btad007_Supplementary_Data

## Data Availability

No new data were generated or analysed in support of this research. *Conflict of Interest*: none declared.
