# Supplementary material for: UPP2: fast and accurate alignment of datasets with fragmentary sequences
Source: Bioinformatics. 2023 Jan 10;39(1):btad007. doi: 10.1093/bioinformatics/btad007 (PMC9846425; doi:10.1093/bioinformatics/btad007)
Supplement: btad007_Supplementary_Data [file btad007_supplementary_data.pdf]

UPP2: Supplementary Materials

Minhyuk Park[1] Stefan Ivanovic[1] Gillian Chu[1] Chengze Shen[1]
Tandy Warnow[1*]

[1]Department of Computer Science, University of Illinois Urbana-Champaign, Champaign, IL 61820
*To whom correspondence should be addressed.

Contents

S1 Additional Details about UPP and UPP2 3

S2 Adjusted bit-scores 4

S3 Method Versions and Commands 5

S4 Dataset properties 9

S5 Additional Results 20
S5.1 Experiment 1 . . . 20
S5.2 Experiment 2a . . . 23
S5.3 Experiment 2c . . . 26
S5.4 Experiment 2b: Results on the 7 smallest CRW datasets . . . 29
S5.5 Other experiments . . . 33

S6 Information about MSA failures 36
S6.1 RNA (CRW) Datasets . . . 36
S6.2 Homfam Datasets . . . 36

S7 Backbone Query Split Algorithm Details 37

List of Figures

S1 Sequence length histogram for 16S.3 biological dataset . . . 10
S2 Sequence length histogram for 16S.T biological dataset . . . 10
S3 Sequence length histogram for 16S.B.ALL biological dataset . . . 11
S4 Sequence length histogram for 16S.A biological dataset . . . 11
S5 Sequence length histogram for 16S.C biological dataset . . . 12
S6 Sequence length histogram for 16S.M biological dataset . . . 12
S7 Sequence length histogram for 23S.A biological dataset . . . 13
S8 Sequence length histogram for 23S.C biological dataset . . . 13
S9 Sequence length histogram for 23S.M biological dataset . . . 14
S10 Sequence length histogram for 5S.3 biological dataset . . . 14
S11 Sequence length histogram for Homfam PDZ biological dataset . . . 15
S12 Sequence length histogram for Homfam blmb biological dataset . . . 15
S13 Sequence length histogram for Homfam p450 biological dataset . . . 16
S14 Sequence length histogram for Homfam adh biological dataset . . . 16
S15 Sequence length histogram for Homfam aat biological dataset . . . 17

S16 Sequence length histogram for Homfam rrm biological dataset . . . 17
S17 Sequence length histogram for Homfam Acetyltransf biological dataset . . . 18
S18 Sequence length histogram for Homfam sdr biological dataset . . . 18
S19 Sequence length histogram for Homfam zf-CCHH biological dataset . . . 19
S20 Sequence length histogram for Homfam rvp biological dataset . . . 19
S21 Experiment 1: Impact of adjusted bit-score and decomposition size . . . 20
S22 Experiment 1: Impact of backbone alignment and stopping rule . . . 21
S23 Experiment 1: Comparison of search strategies in UPP2 . . . 22
S24 Experiment 2a: Runtime on simulated HF datasets . . . 23
S25 Experiment 2a: Evaluation of MSA methods on simulated HF datasets . . . 24
S26 Experiment 2c: UPP2 compared to other MSA methods on individual Homfam datasets . . 27
S27 Experiment 2c: Average performance of UPP2 compared to other MSA methods on Homfam datasets . . . 28
S28 Experiment 2b: MAFFT variants on small to medium RNA datasets . . . 30
S29 Experiment 2: UPP variants compared to other MSA methods on small to medium RNA datasets . . . 31
S30 Experiment 2: UPP variants compared to other MSA methods on small to medium RNA datasets . . . 32
S31 Extra Experiment: Evaluation of MSA methods (alignment error and runtime) on simulated datasets without fragmentation . . . 34
S32 Extra Experiment: Runtime of UPP2 against UPP(MAGUS)+adj on simulated datasets without fragments . . . 35
S33 Extra Experiment: Impact of Different Full-length Sampling Approaches . . . 35

List of Tables

S1 ROSE, RNASim, and CRW dataset properties . . . 9
S2 Homfam dataset properties . . . 9
S3 Experiment 2a: P-values for the ntatistical tests in Figure 4 . . . 25
S4 Homfam SPFN error with reported T-COFFEE . . . 26
S5 Homfam Runtime on individual datasets . . . 26
S6 Experiment 2b: UPP variants compared to other MSA methods on small to medium RNA datasets . . . 29

S1 Additional Details about UPP and UPP2

Here we describe some additional details about UPP and UPP2 that were not provided in the main paper due to space limitations.

UPP2 uses the same *hmmsearch* parameters as UPP, such as setting an extremely high e-value cut-off and turning off all filters, in order to increase the probability of assigning each query sequence to an HMM. If a query sequence has no bit-score for a given HMM, then the bit-score will default to zero. If there are ties after computing the adjusted bit-scores, then the tie will be broken randomly, thus ensuring that every query sequence is assigned to a unique HMM. Then, each query sequence is added into the subset alignment for the selected HMM using *hmmalign*. By default, *hmmalign* outputs the outer edge unaligned residues as lower case characters, which are present in the output alignment of UPP and UPP2 as well. Following UPP's procedures, unaligned residues from different sequences are collapsed into a single column in order to save space.

S2 Adjusted bit-scores

One of the differences between UPP2 and the earlier version (available in the original github page) is the use of adjusted bit-scores instead of "raw" bit-scores; this modification is published in [3], and provided here to be self-contained.

The bit-score of a query sequence given a HMMER HMM is $\log_2 \frac{P(q|H)}{P(q|H_0)}$ where H is the HMM, q is the query sequence, and H_0 is the null model, or the random model. Using Bayes' theorem, we arrive at the probability of H_i generating sequence q as follows.

$$\begin{aligned} P(H_i|q) &= \frac{P(q|H_i) \cdot P(H_i)}{P(q)} \\ &= \frac{P(q|H_i) \cdot P(H_i)}{\Sigma_{j=1}^{n} P(q|H_j) \cdot P(H_j)} \end{aligned}$$

where n is the number of HMMs ($H_i ... H_n$).

If we assume that the more sequences the HMM is trained on the more likely the HMM is to output a sequence, then we can transform the above into the following.

$$\begin{aligned} P(H_i|q) &= \frac{P(q|H_i) \cdot \frac{s_i}{S}}{\Sigma_{j=1}^{n} P(q|H_j) \cdot \frac{s_j}{S}} \\ &= \frac{1}{\Sigma_{j=1}^{n} \frac{P(q|H_j) \cdot s_j}{P(q|H_i) \cdot s_i}} \\ &= \frac{1}{\Sigma_{j=1}^{n} 2^{\log_2 \frac{P(q|H_j) \cdot s_j}{P(q|H_i) \cdot s_i}}} \end{aligned}$$

where s_i is the number of sequences that HMM H_i was trained on and S is the total number of sequences that the HMMs were trained on.

From the definition of bit-scores, we can derive the following:

$$\begin{aligned} BS(H_j) - BS(H_i) &= \log_2 \frac{P(q|H_j)}{P(q|H_0)} - \log_2 \frac{P(q|H_i)}{P(q|H_0)} \\ &= \log_2 \frac{P(q|H_j)}{P(q|H_i)} \end{aligned}$$

So

$$\begin{aligned} P(H_i|q) &= \frac{1}{\Sigma_{j=1}^{n} 2^{\log_2 \frac{P(q|H_j) \cdot s_j}{P(q|H_i) \cdot s_i}}} \\ &= \frac{1}{\Sigma_{j=1}^{n} 2^{BS(H_j) - BS(H_i) + \log_2 \frac{s_j}{s_i}}} \end{aligned}$$

As shown in [3], adjusted bit-scores are always between 0 and 1, and can be interpreted as the probability that the given HMM generates the given query sequence. In particular, the sum, across all the HMMs in the ensemble, is always 1.

S3 Method Versions and Commands

FastSP

- Version: 1.7.1
- Availability: `https://github.com/smirarab/FastSP`
- Note: -ml and -mlr flags are omitted for MAFFT alignments since MAFFT only outputs lowercase characters
- Command:

```
java -jar FastSP.jar -ml -mlr -r <REFERENCE ALIGNMENT> -e <ESTIMATED ALIGNMENT>
```

MAFFT-linsi

- Version: 7.487
- Availability: `https://mafft.cbrc.jp/alignment/software/`
- Command:

```
linsi --thread 16 <SEQUENCE FILE> 1> <OUTPUT>/mafft.fasta
```

MAFFT auto

- Version: 7.487
- Availability: `https://mafft.cbrc.jp/alignment/software/`
- Command:

```
mafft --auto --thread 16 <SEQUENCE FILE> 1> <OUTPUT>/mafft.fasta
```

MAFFT-ginsi

- Version: 7.487
- Availability: `https://mafft.cbrc.jp/alignment/software/`
- Command:

```
mafft-ginsi --thread 16 <SEQUENCE FILE> 1> <OUTPUT>/mafft.fasta
```

MAFFT-qinsi

- Version: 7.487
- Availability: `https://mafft.cbrc.jp/alignment/software/`
- Command:

```
mafft-qinsi --thread 16 <SEQUENCE FILE> 1> <OUTPUT>/mafft.fasta
```

MAFFT-xinsi

- Version: 7.487
- Availability: `https://mafft.cbrc.jp/alignment/software/`
- Command:

```
mafft-xinsi --thread 16 <SEQUENCE FILE> 1> <OUTPUT>/mafft.fasta
```

MUSCLE

- Version: 3.8.31
- Availability: `https://www.drive5.com/muscle/`
- Command:

```
muscle -in <SEQUENCE FILE> -out <OUTPUT>/muscle.fasta
```

ClustalOmega

- Version: 1.2.4
- Availability: `http://www.clustal.org/omega/`
- Command:

```
clustalo --threads=16 --in <SEQUENCE FILE> --out <OUTPUT>/clustalo.fasta
```

MAGUS

- Commit id: 95522ec9539575189a0a2f90baaf81cbde480034
- Availability: `https://github.com/vlasmirnov/MAGUS`
- Command:

```
python MAGUS/magus.py -d <OUTPUT> -i <SEQUENCE FILE> -o <OUTPUT>/magus.fasta
```

T-COFFEE

- Version: Version 13.45.0.4846264
- Availability: `http://www.tcoffee.org/`
- Command:

```
t_coffee -thread=16 -reg -seq <SEQUENCE FILE> -outfile <OUTPUT>/t_coffee.fasta
```

PASTA

- Version: PASTA v1.9.0
- Availability: `https://github.com/smirarab/PASTA`
- Command:

  ```
  python run_pasta.py -i <SEQUENCE FILE> --num-cpus 16 -o <OUTPUT> --temporaries \
  <OUTPUT>
  ```

UPP and UPP2

- Commit ID: 05591949d78f9e98f51c000f119c4b191f2696ef
- Availability: `https://github.com/gillichu/sepp`
- Note: By setting decomp_only and bitscore_adjust as True and leaving hier_upp and early_stop as False, we are able to replicate UPP's algorithm with adjusted bit-scores.
- Command:

```
python run_upp.py -c <CONFIG FILE>
```

UPP2 Config

```
[commandline]
sequence_file=<QUERY SEQUENCES>
alignment=<BACKBONE FASTA>
tree=<BACKBONE TREE>
backboneSize=<BACKBONE SIZE>
alignmentSize=<DECOMPOSITION SIZE>
molecule=<dna/rna/amino>
cpu=16
tempdir=<TEMP DIRECTORY>
outdir=<OUTPUT DIRECTORY>

[upp2]
decomp_only=True
bitscore_adjust=True
hier_upp=False
early_stop=False
```

UPP2-Hierarchical Config

```
[commandline]
sequence_file=<QUERY SEQUENCES>
alignment=<BACKBONE FASTA>
tree=<BACKBONE TREE>
backboneSize=<BACKBONE SIZE>
alignmentSize=<DECOMPOSITION SIZE>
molecule=<dna/rna/amino>
cpu=16
tempdir=<TEMP DIRECTORY>
outdir=<OUTPUT DIRECTORY>

[upp2]
decomp_only=True
bitscore_adjust=True
hier_upp=True
early_stop=False
```

UPP2 (UPP2-EarlyStop) Config

```
[commandline]
sequence_file=<QUERY SEQUENCES>
alignment=<BACKBONE FASTA>
tree=<BACKBONE TREE>
```

```
backboneSize=<BACKBONE SIZE>
alignmentSize=<DECOMPOSITION SIZE>
molecule=<dna/rna/amino>
cpu=16
tempdir=<TEMP DIRECTORY>
outdir=<OUTPUT DIRECTORY>

[upp2]
decomp_only=True
bitscore_adjust=True
hier_upp=True
early_stop=True
```

S4 Dataset properties

Table S1: ROSE, RNASim, and CRW dataset properties. We show the average and maximum p-distances (normalized Hamming distances) and number of sequences in each of the study datasets. The ROSE and RNASim datasets are studied in two versions: unmodified (i.e., without fragmentation) and high-fragmentation (HF, where 50% of the sequences are shortened to approximately 25% of the original median sequence length). Here we show the empirical properties of the unmodified versions of these datasets.

Name	Sim/Bio	# Sequences	avg. p-dist.	max. p-dist.
1000S1	Sim	1000	0.694	0.768
1000S2	Sim	1000	0.693	0.768
1000S3	Sim	1000	0.686	0.763
1000S4	Sim	1000	0.501	0.608
1000S5	Sim	1000	0.498	0.611
1000M1	Sim	1000	0.695	0.769
1000M2	Sim	1000	0.684	0.762
1000M3	Sim	1000	0.660	0.741
1000M4	Sim	1000	0.495	0.606
1000M5	Sim	1000	0.499	0.602
1000L1	Sim	1000	0.695	0.769
1000L2	Sim	1000	0.696	0.769
1000L3	Sim	1000	0.687	0.763
1000L4	Sim	1000	0.500	0.608
1000L5	Sim	1000	0.496	0.606
RNASim1000	Sim	1000	0.411	0.609
16S.3	Bio	6323	0.315	0.833
16S.T	Bio	7350	0.345	0.901
16S.B.ALL	Bio	27643	0.210	0.769
16S.A	Bio	594	0.185	0.673
16S.C	Bio	320	0.157	1.000
16S.M	Bio	805	0.359	0.768
23S.A	Bio	214	0.293	0.667
23S.C	Bio	374	0.143	0.750
23S.M	Bio	254	0.380	0.695
5S.3	Bio	5507	0.418	1.000

Table S2: **Homfam dataset properties**. We show estimates of the average and maximum p-distances (normalized Hamming distances) and number of sequences in each of the Homfam datasets. 1000 sequences were sampled from the MAGUS estimated alignments of each dataset to compute the p-distances

Name	Sim/Bio	# Sequences	avg. p-dist.	max. p-dist.
PDZ	Bio	14,950	0.755	1.0
blmb	Bio	17,200	0.802	1.0
p450	Bio	21,013	0.791	1.0
adh	Bio	21,331	0.779	1.0
aat	Bio	25,100	0.810	1.0
rrm	Bio	27,610	0.763	1.0
Acetyltransf	Bio	46,285	0.801	1.0
sdr	Bio	50,157	0.747	1.0
zf-CCHH	Bio	88,345	0.635	0.882
rvp	Bio	93,681	0.140	0.805

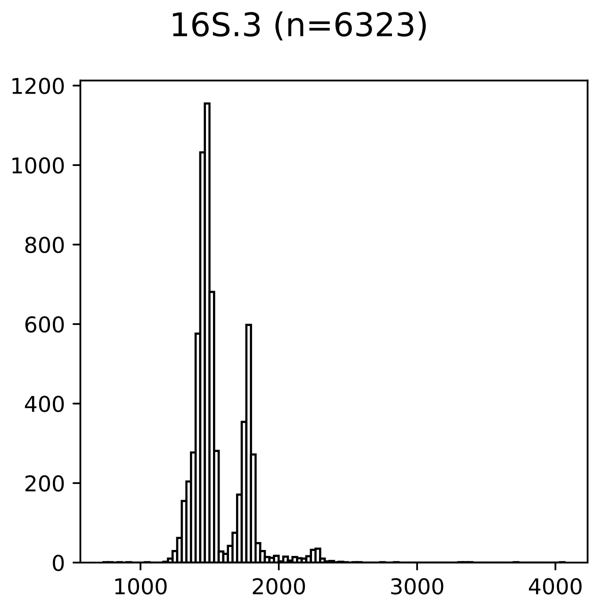

Figure S1: **Sequence length histogram for 16S.3 biological dataset** The sequence length histogram for the 16S.3 indicates at least two peaks with some sequences with length 1000 and others over 2000.

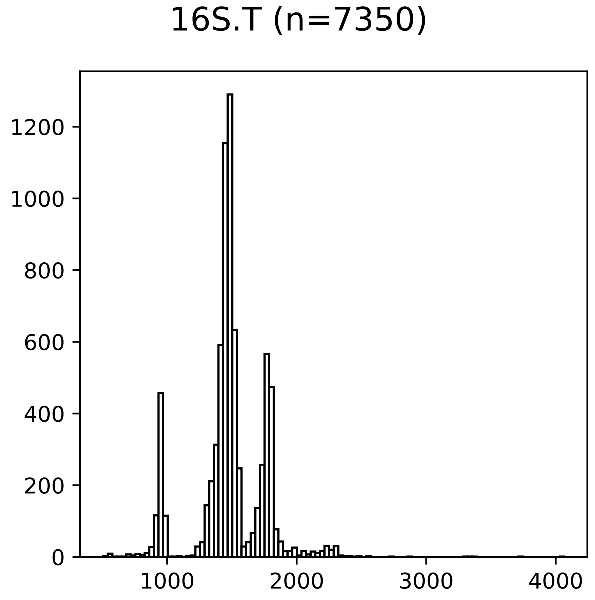

Figure S2: **Sequence length histogram for 16S.T biological dataset** The sequence length histogram for the 16S.T dataset indicates at least three peaks with one of the peaks at around 1000 and another peak closer to 2000.

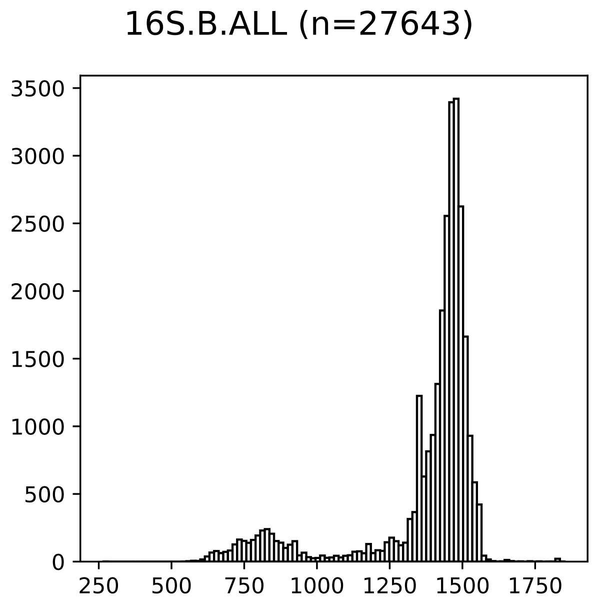

Figure S3: **Sequence length histogram for 16S.B.ALL biological dataset** The sequence length histogram for the 16S.B.ALL dataset shows moderate number of sequences with length shorter than 1250 and a peak at a little below 1500.

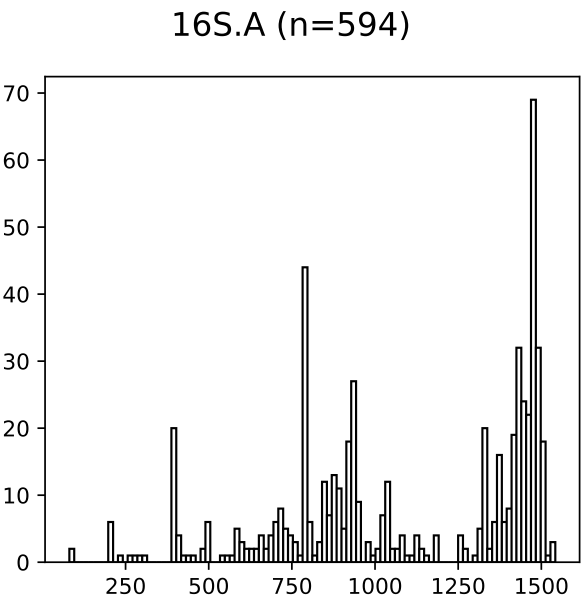

Figure S4: **Sequence length histogram for 16S.A biological dataset** The sequence length histogram for the 16S.A dataset shows a peak around 1450 and another peak around 850.

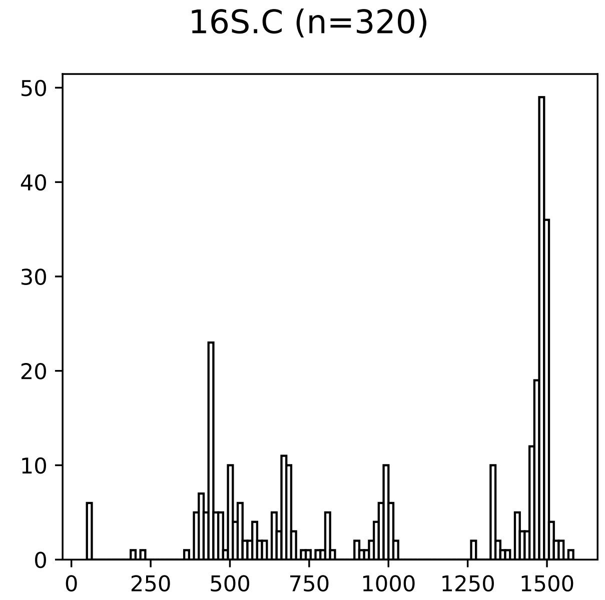

Figure S5: **Sequence length histogram for 16S.C biological dataset** The sequence length histogram for the 16S.C dataset shows a peak around 1500 and another gentler peak around 550.

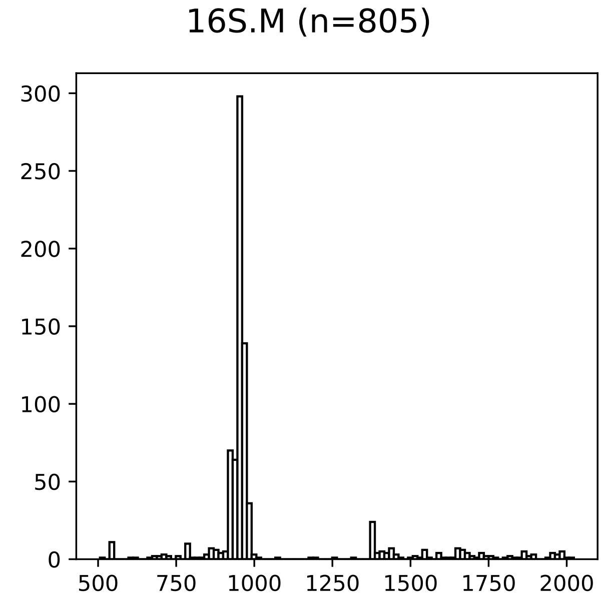

Figure S6: **Sequence length histogram for 16S.M biological dataset** The sequence length histogram for the 16S.M dataset shows a tall peak around 950 with sequences ranging from around 500 to 2000.

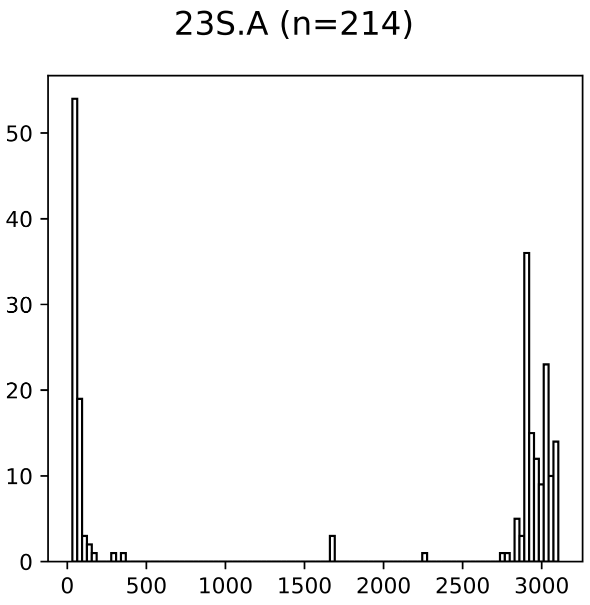

Figure S7: **Sequence length histogram for 23S.A biological dataset** The sequence length histogram for the 23S.A dataset shows a peak near 1 and another peak around 3000.

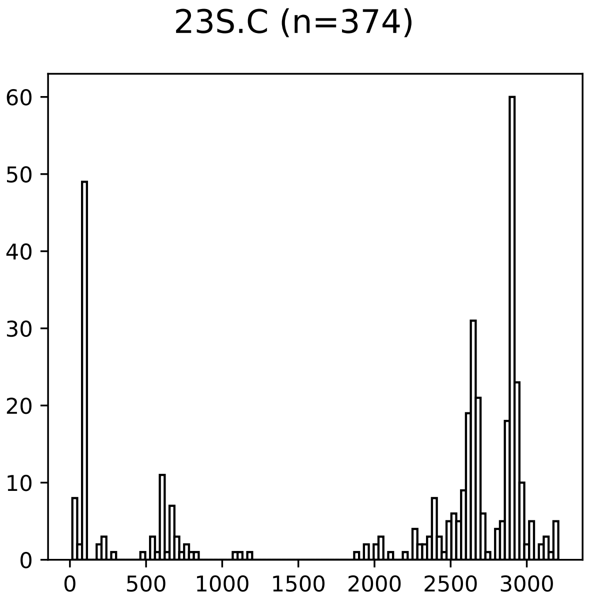

Figure S8: **Sequence length histogram for 23S.C biological dataset** The sequence length histogram for the 23S.C dataset shows a wide peak from around 2000 to 3250 and another wide peak from zero to about 750.

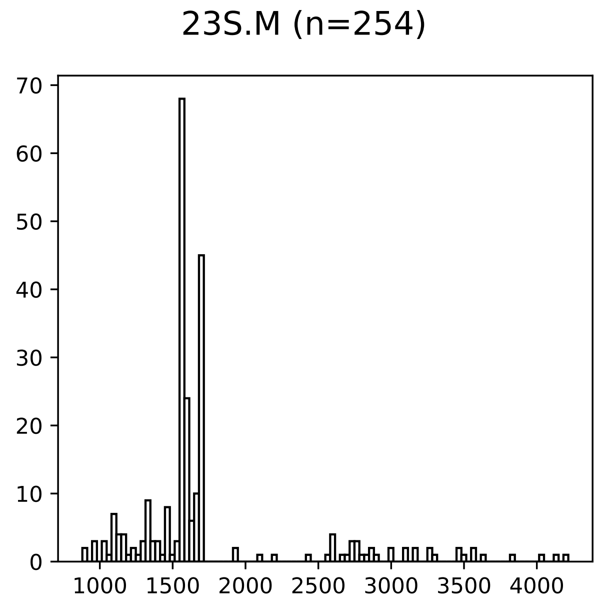

Figure S9: **Sequence length histogram for 23S.M biological dataset** The sequence length histogram for the 23S.M dataset shows a tall peak around 1500 with sequences ranging from about 500 to 4250.

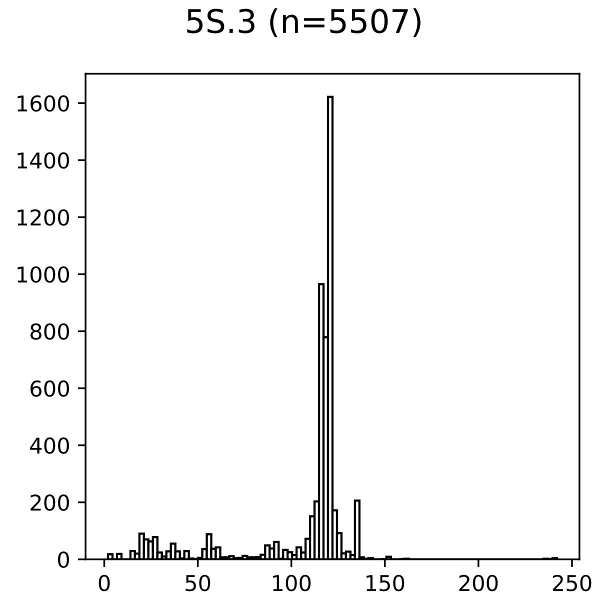

Figure S10: **Sequence length histogram for 5S.3 biological dataset** The sequence length histogram for the 5S.3 dataset shows a tall peak around 125 with some shorter sequences ranging from 0 to 125 and very few sequences longer than 150.

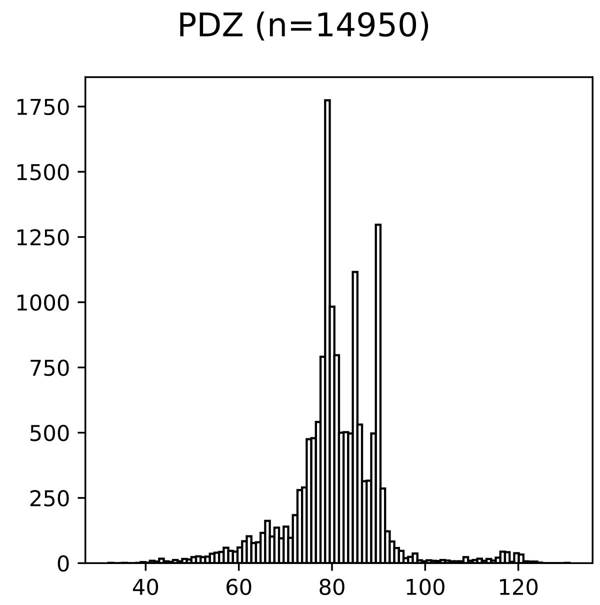

Figure S11: **Sequence length histogram for PDZ Homfam biological dataset** The sequence length histogram for the PDZ Homfam dataset shows sequences as short as 40 base pairs long as well as sequences as long as 120 base pairs long.

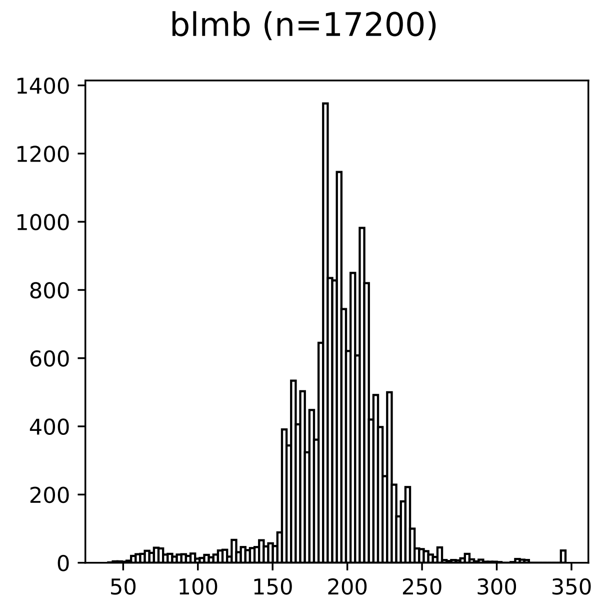

Figure S12: **Sequence length histogram for blmb Homfam Biological Dataset** The sequence length histogram for the blmb Homfam dataset shows that the majority of sequence lengths lie between 150 and 250 with some sequence lengths uniformly spread from 50 to 150.

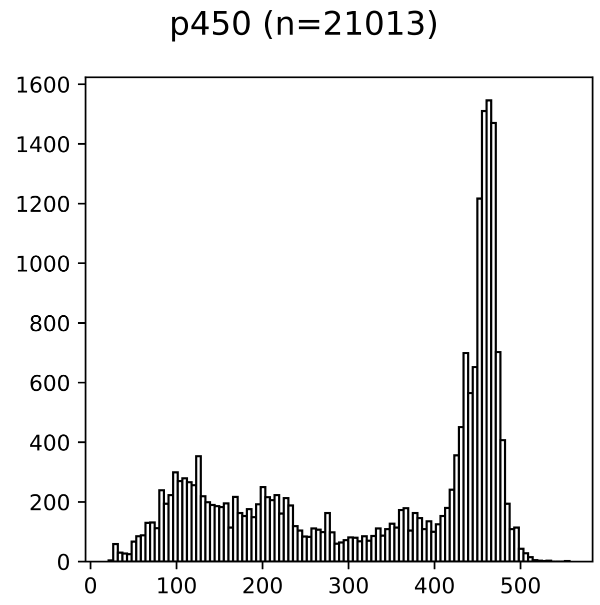

Figure S13: **Sequence length histogram for p450 Homfam Biological Dataset** The sequence length histogram for the p450 Homfam dataset shows a tall peak at around 470 with many sequences below 400 and a smaller peak at 100.

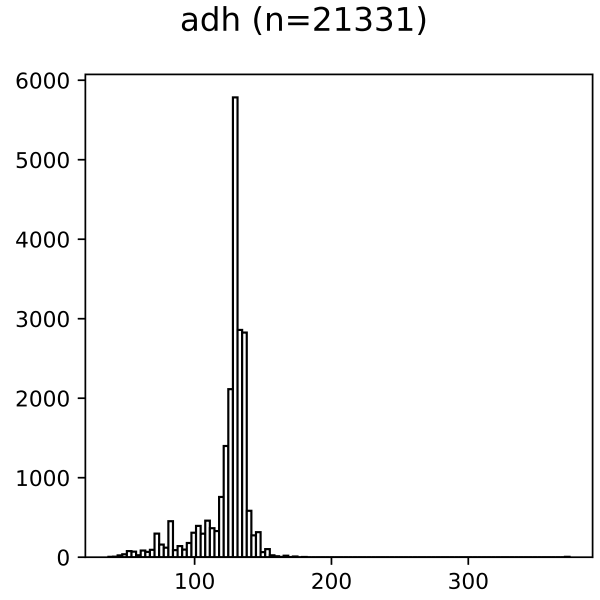

Figure S14: **Sequence length histogram for adh Homfam Biological Dataset** The sequence length histogram for the adh Homfam dataset shows a peak at around 130 with some sequences shorter than 100.

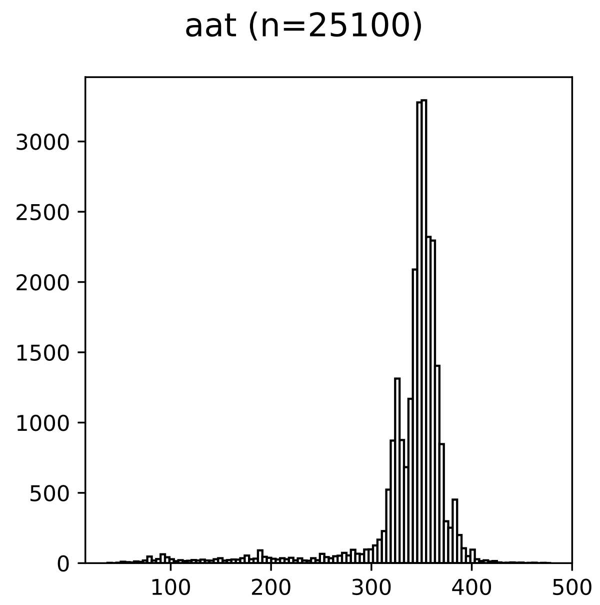

Figure S15: **Sequence length histogram for aat Homfam Biological Dataset** The sequence length histogram for the aat Homfam dataset shows a peak at around 350 with some sequences uniformly distributed from below 100 to 300.

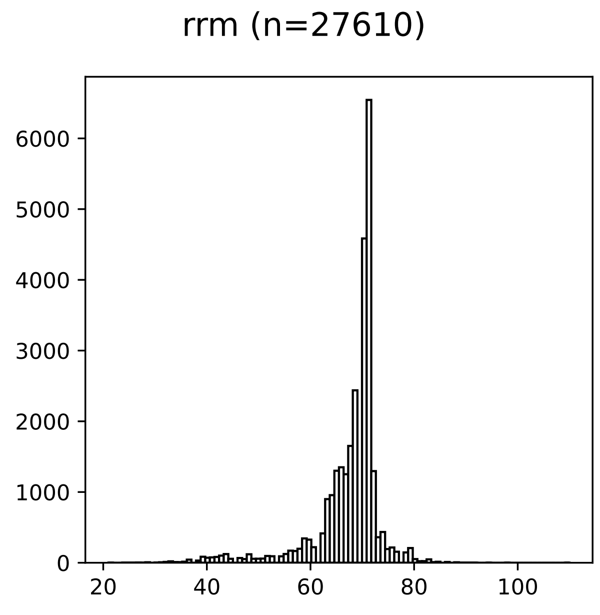

Figure S16: **Sequence length histogram for rrm Homfam Biological Dataset** The sequence length histogram for the rrm Homfam dataset shows a peak at around 70 with shorter sequences ranging from about 40 to 60.

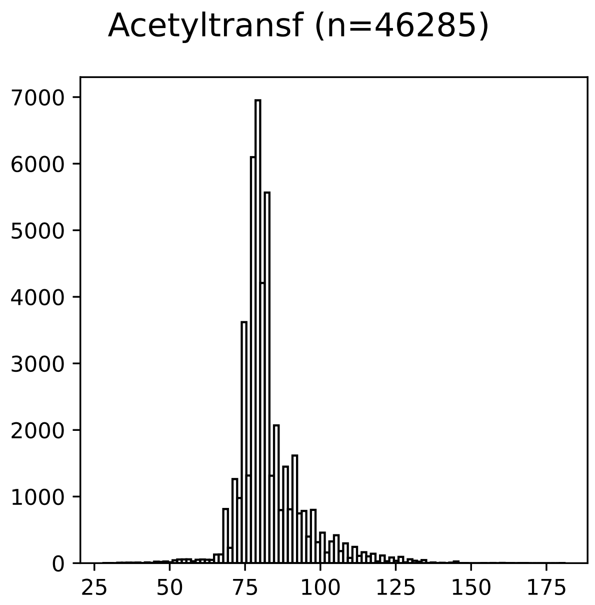

Figure S17: **Sequence length histogram for Acetyltransf Homfam Biological Dataset** The sequence length histogram for the Acetyltransf Homfam dataset shows a peak at around 80 with more sequences to the right of the peak but less so to the left of the peak.

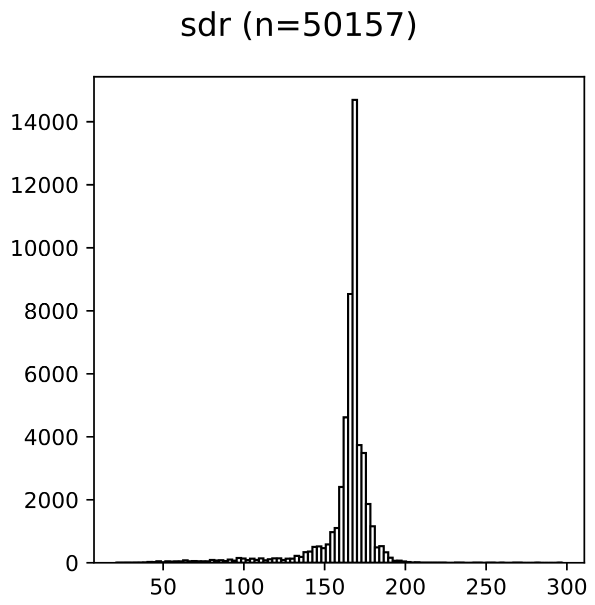

Figure S18: **Sequence length histogram for sdr Homfam Biological Dataset** The sequence length histogram for the sdr Homfam dataset shows a peak at around 170 with some sequences ranging from around 50 to 150.

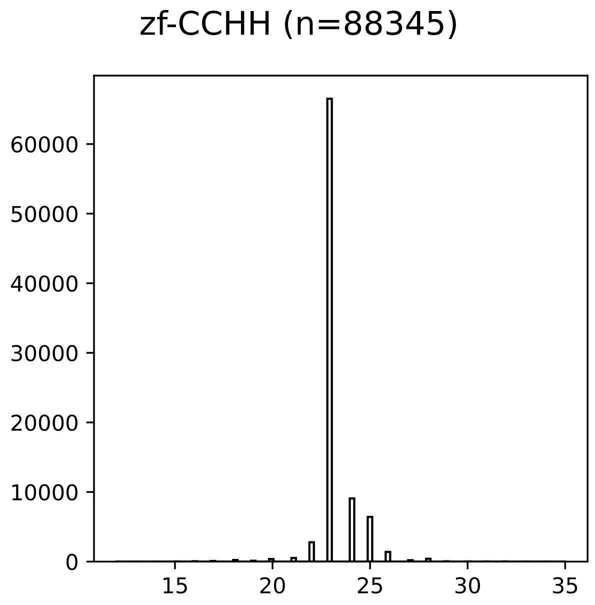

Figure S19: **Sequence length histogram for zf-CCHH Homfam Biological Dataset** The sequence length histogram for the zf-CCHH Homfam dataset shows the majority of the sequences between 20 and 25.

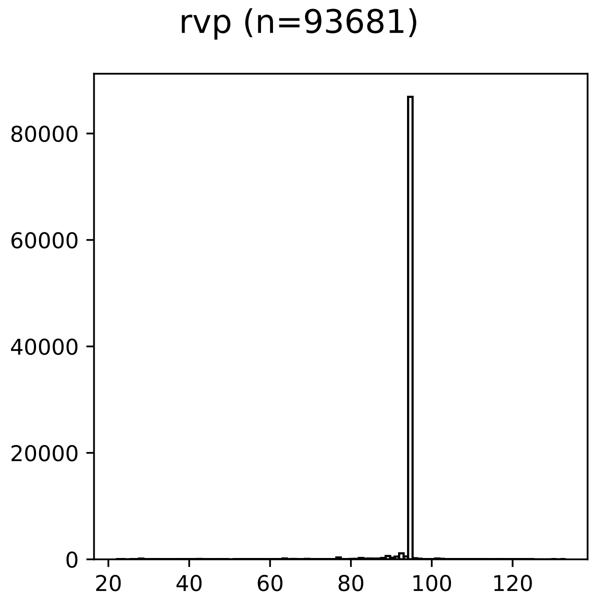

Figure S20: **Sequence length histogram for rvp Homfam biological dataset**] The sequence length histogram for the rvp dataset shows the majority of the sequences between 80 and 100 with most sequences falling under the same bin at around 95.

S5 Additional Results

S5.1 Experiment 1

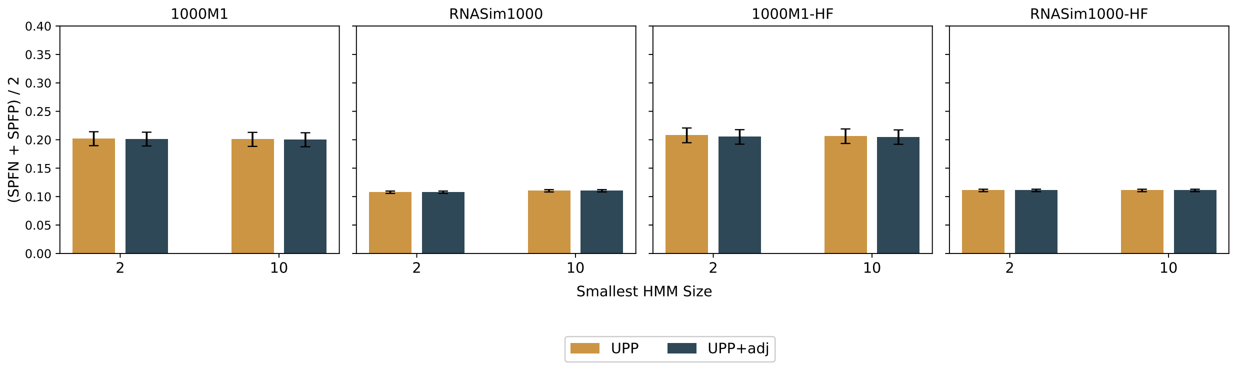

Figure S21: **Experiment 1: Impact of Adjusted Bit-score and Decomposition Size (size of smallest subsets) on Alignment Error** Both UPP and UPP+adj in this Figure uses PASTA backbone alignments. UPP uses the raw bit-scores while UPP+adj uses the adjusted bit-scores. Both methods perform an all-against-all search of HMMs to query sequences. Each subfigure shows two values for z, the size of the smallest subset within the decomposition strategy (i.e., decomposition size). The bar indicates the mean while the error bars indicate standard error.

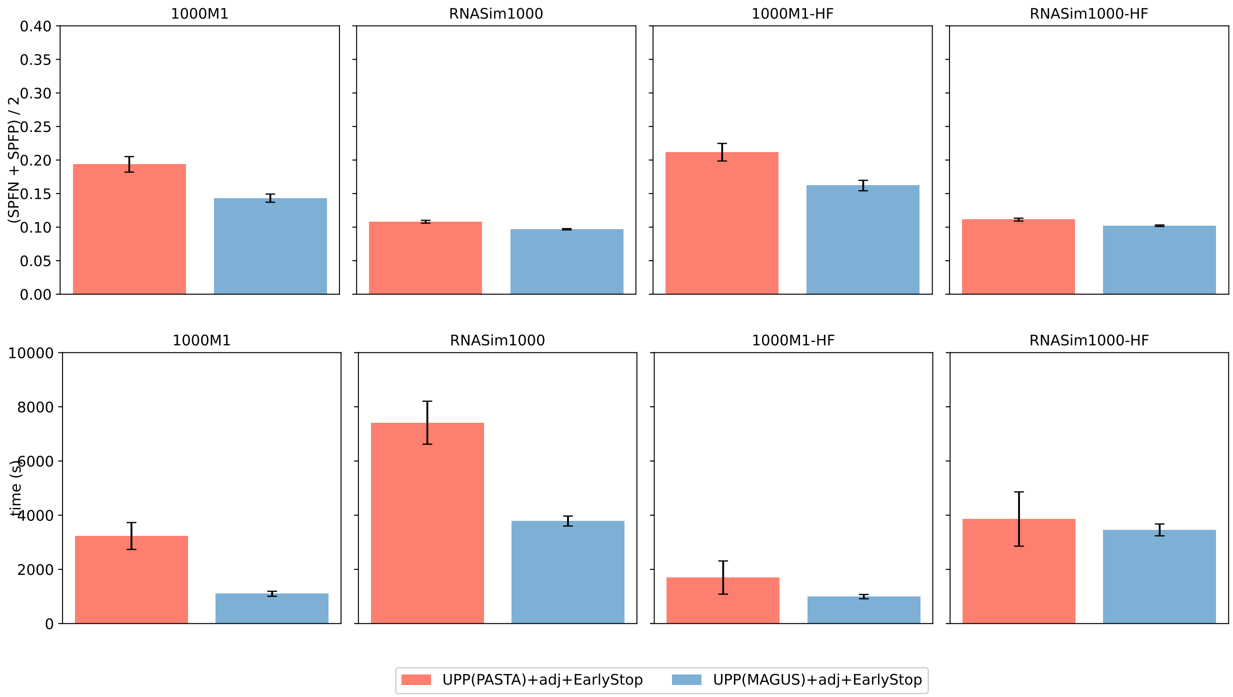

Figure S22: **Experiment 1: Impact of choice of backbone alignment method (MAGUS vs. PASTA) and Stopping Rule on alignment accuracy and total runtime** 1000M1 has 19 replicates, RNASim1000 has 20 replicates, 1000M1-HF has 19 replicates, and RNASim1000-HF has 20 replicates. The means are shown with error bars indicating standard error for alignment error and standard deviation for running time.

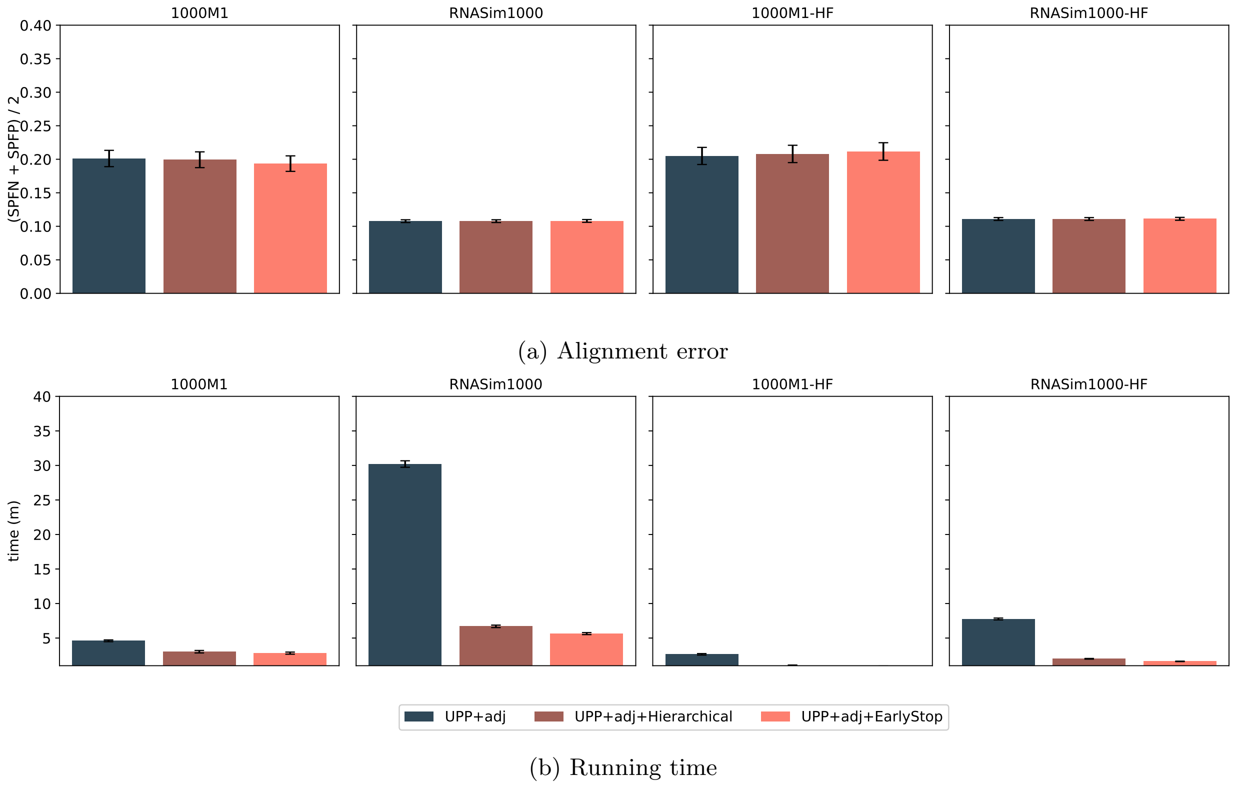

Figure S23: **Experiment 1: Impact of Hierarchical and EarlyStop Search Strategies on UPP(PASTA)+adj Alignment Error and Runtime** In this figure, UPP+adj uses the PASTA backbone alignment on full-length sequences, sets $z = 2$, and uses adjusted bit-scores. z refers to the decomposition size of UPP. The runtime reported here does not include the time to compute the backbone alignment and tree. The means are shown with error bars indicating standard error for alignment error and standard deviation for running time.

S5.2 Experiment 2a

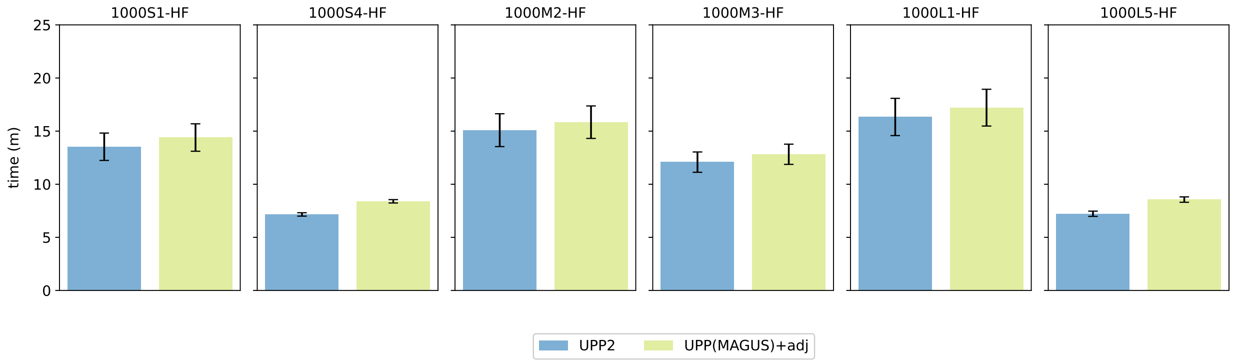

Figure S24: **Experiment 2a: Runtime Comparison of UPP2 and UPP(MAGUS)+adj on Simulated High Fragmentary Datasets** UPP(MAGUS)+adj and UPP2 both use MAGUS backbone alignments, FastTree backbone trees, and adjusted bit-scores; they differ in their search strategies (EarlyStop vs. all-against-all). All datasets have 20 replicates each. The means are shown with error bars indicating standard deviation for running time.

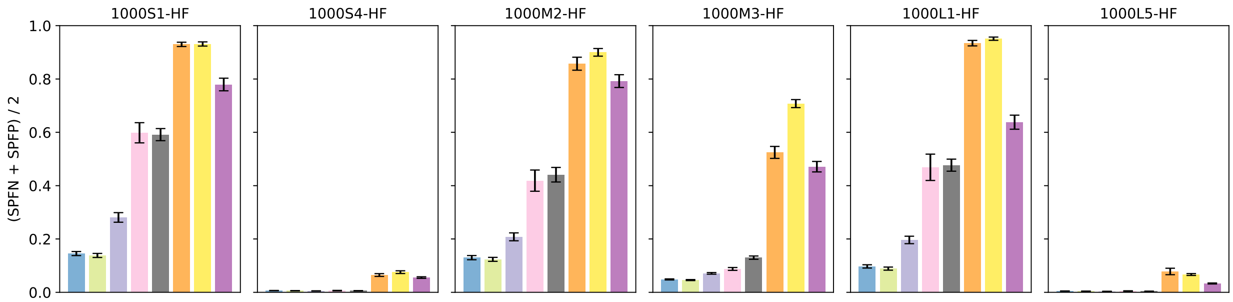

(a) Average alignment error

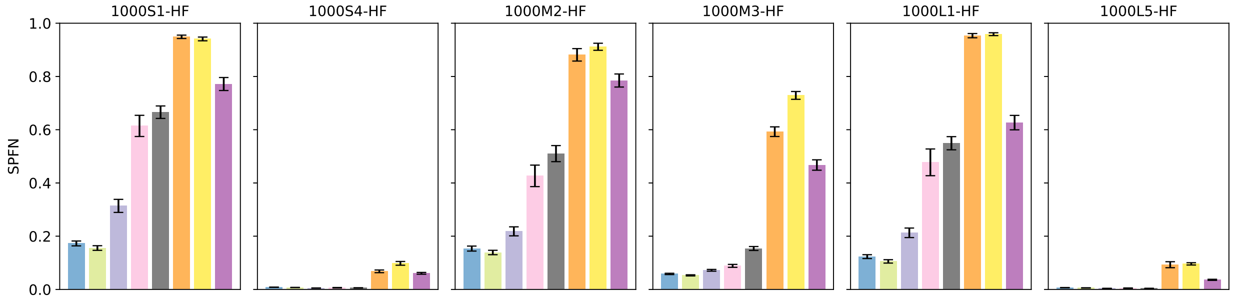

(b) SPFN alignment error

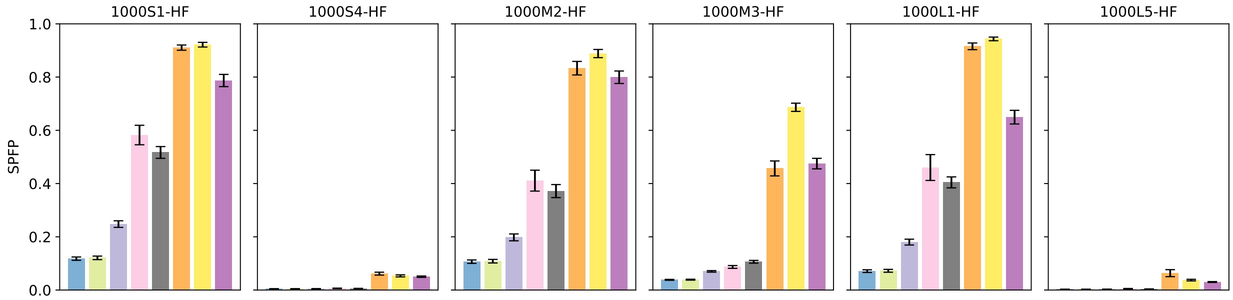

(c) SPFP alignment error

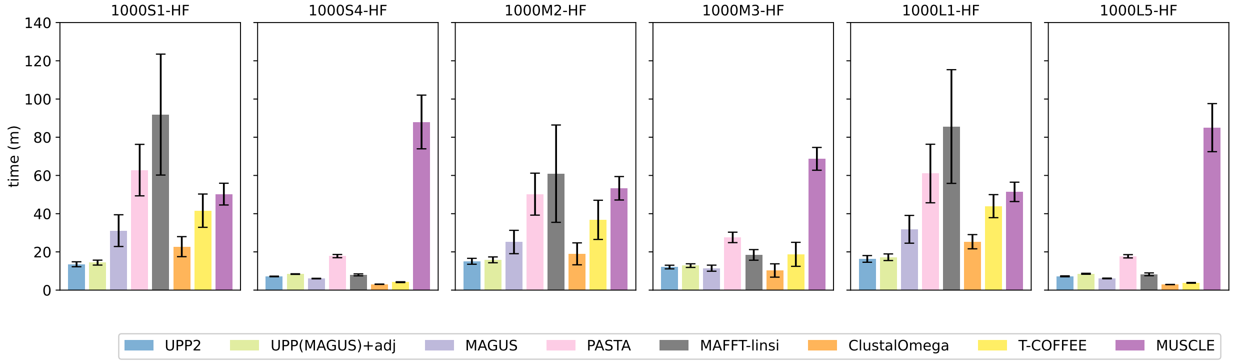

(d) running time

Figure S25: **Experiment 2a: UPP2 Compared to Other MSA Methods on High Fragmentary Simulated Datasets** We show alignment error and runtime of UPP2 (i.e., UPP(MAGUS)+adj+EarlyStop) compared to other alignment methods. All methods except T-COFFEE and MUSCLE were run in their default modes and with 16 threads, when possible. T-COFFEE was run using the regressive mode and MUSCLE was limited to 2 iterations. All datasets have 20 replicates each. The means are shown with error bars indicating standard error for alignment error and standard deviation for running time.

Table S3: **P-values for the Statistical Tests in Figure 4** We show the p-values of independent two-sample t-tests of UPP2 compared to the next best method (MAGUS). A positive test statistic indicates that the next best method (MAGUS) was more accurate than UPP2 while a negative test statistic indicates that UPP2 was more accurate than the next best method (MAGUS). Note that there are many conditions where the differences are statistically significant (indicated by $p < 0.05$). However, the only cases that were statistically significant and noteworthy (i.e., differences greater than 0.01) are where UPP2 is more accurate than MAGUS.

Name	test statistic	p-value	MAGUS error rate	UPP2 error rate
1000S5-HF	4.55	5.86×10^{-5}	0.002	0.004
1000L4-HF	2.42	2.08×10^{-2}	0.007	0.008
1000M4-HF	2.02	5.13×10^{-2}	0.011	0.013
1000M5-HF	1.52	1.38×10^{-1}	0.006	0.007
1000L5-HF	2.38	2.27×10^{-2}	0.003	0.005
1000S4-HF	2.91	6.16×10^{-3}	0.005	0.006
1000M3-HF	-5.89	9.63×10^{-7}	0.071	0.049
1000S3-HF	-7.52	6.85×10^{-9}	0.118	0.064
1000S2-HF	-8.55	3.47×10^{-10}	0.151	0.078
1000M2-HF	-4.46	7.61×10^{-5}	0.204	0.128
1000L2-HF	-5.12	1.03×10^{-5}	0.141	0.054
1000L1-HF	-6.29	2.83×10^{-7}	0.198	0.099
1000L3-HF	-7.62	5.12×10^{-9}	0.247	0.149
1000S1-HF	-7.62	5.18×10^{-9}	0.271	0.141

S5.3 Experiment 2c

To enable a comparison to T-COFFEE Regressive, we also provide a comparison of SPFN values in Table S4, using results for T-COFFEE Regressive obtained from [2]. MAGUS provides the best accuracy, followed by UPP2 using the ALL setting, and then by MAFFT in auto mode follows. T-COFFEE Regressive and UPP2 with default settings are very close but with a small advantage to T-COFFEE.

Runtime comparisons between UPP2, MAGUS, and MAFFT (run in auto mode) are provided in Table S5.

Table S4: **Homfam Individual Dataset SPFN Error with T-COFFEE Results** The SPFN error of UPP2, MAGUS, MAFFT, and T-COFFEE are shown.

	UPP2	MAGUS	MAFFT	T-COFFEE
Average	0.344	0.285	0.324	0.341
PDZ	0.181	0.214	0.163	0.320
blmb	0.461	0.298	0.447	0.776
p450	0.292	0.263	0.449	0.359
adh	0.653	0.269	0.019	0.014
aat	0.173	0.186	0.274	0.275
rrm	0.251	0.237	0.292	0.275
Acetyltransf	0.522	0.566	0.551	0.549
sdr	0.402	0.398	0.544	0.409
zf-CCHH	0.187	0.210	0.213	0.231
rvp	0.153	0.213	0.288	0.197

Table S5: **Homfam Individual Dataset Runtimes** The runtime of UPP2, MAGUS, and MAFFT (in minutes) are shown. Results for T-Coffee are not shown since we were unable to run T-COFFEE, and runtimes reported on other computational environments for T-COFFEE cannot be compared to runtimes reported here.

	UPP2	MAGUS	MAFFT
Average	42.1	39.0	3.7
PDZ	3.7	9.2	0.6
blmb	19.6	33.3	1.6
p450	29.7	108.2	3.7
adh	6.1	17.3	0.8
aat	129.0	58.6	3.2
rrm	39.8	11.2	0.8
Acetyltransf	145.1	29.8	3.1
sdr	120.0	47.1	7.4
zf-CCHH	37.5	19.9	3.9
rvp	30.3	55.0	11.4

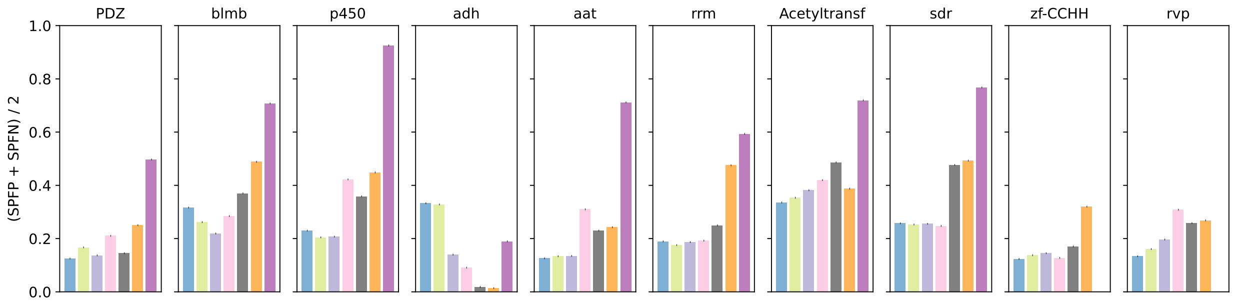

(a) Average alignment error

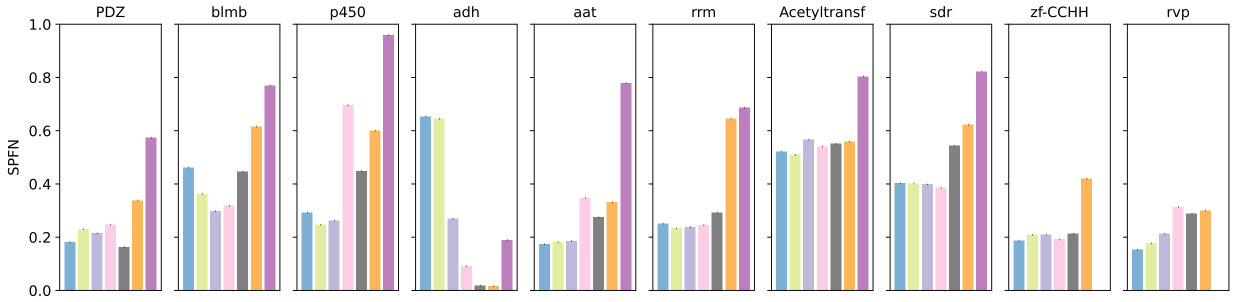

(b) SPFN alignment error

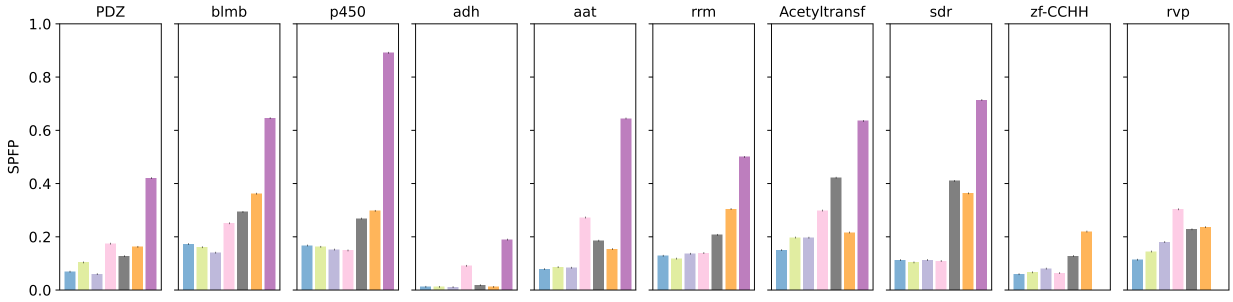

(c) SPFP alignment error

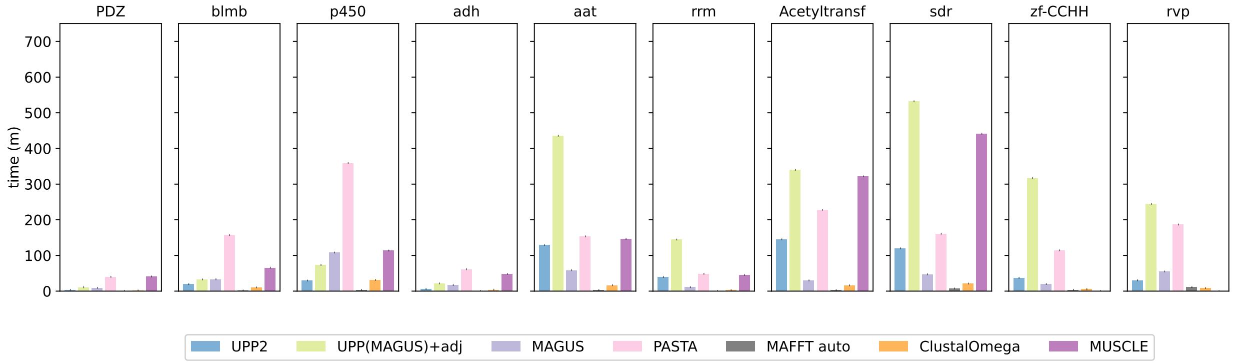

(d) Running time

Figure S26: **Experiment 2c: Alignment Error and Runtime of UPP2 Compared to Other MSA Methods on Individual Homfam Datasets** Of the full-length sequences, 1000 sequences for the smallest four datasets and 10,000 sequences for the six largest datasets were chosen for the backbone. UPP2 and UPP(MAGUS)+adj used a decomposition size of 2 in all datasets. MUSCLE could not run on zf-CCHH and rvp, but the remaining benchmark methods in the legend completed on all the datasets. The number of sequences are as follows: PDZ (14,950), blmb (17,200), p450 (21,013), adh (21,331), aat (25,100), rrm (27,610), Acetyltransf (46,285), sdr (50,157), zf-CCHH (88,345), rvp (93,681).

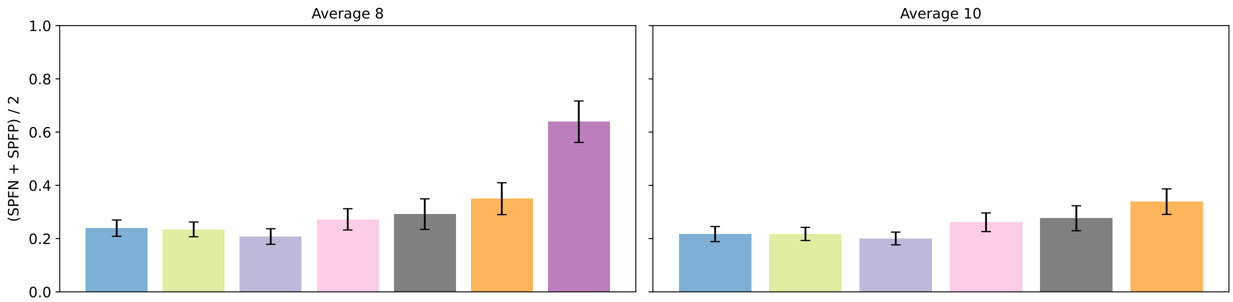

(a) Alignment error

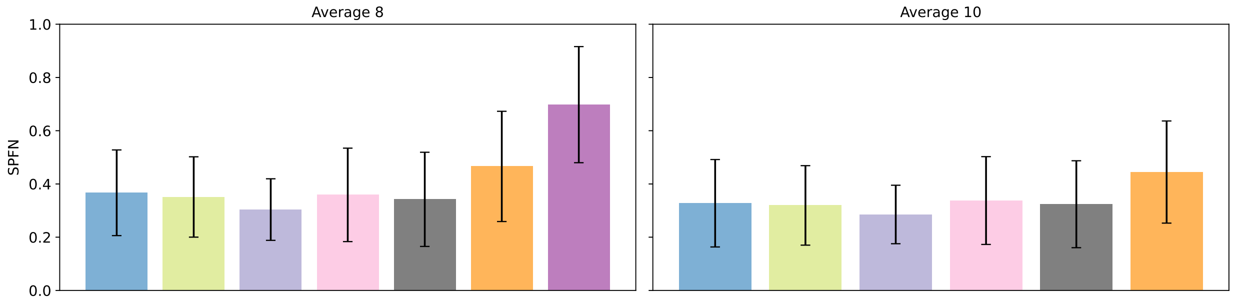

(b) SPFN alignment error

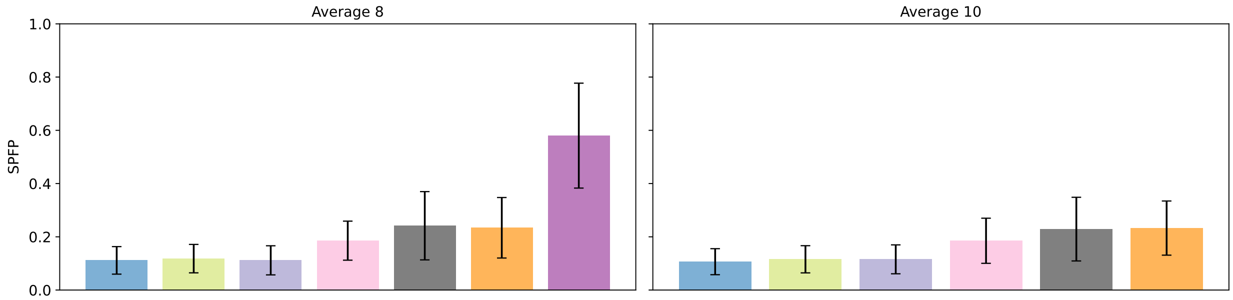

(c) SPFP alignment error

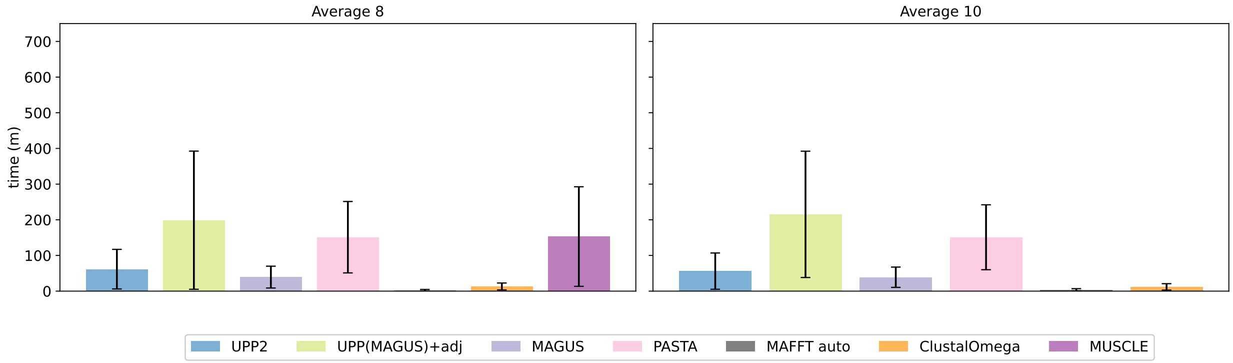

(d) Running time

Figure S27: **Experiment 2c: Average Alignment error and Runtime of UPP2 Compared to Other MSA Methods on the Homfam Datasets** From the full-length sequences, 1000 sequences were chosen for the backbone in the four smallest datasets while 10,000 sequences were chosen for the backbone. UPP2 and UPP(MAGUS)+adj used a decomposition size of 2 in all datasets. MUSCLE could not run on zf-CCHH and rvp, but the remaining benchmark methods in the legend completed on all the datasets. The results on eight datasets, excluding the two datasets which MUSCLE could not run on, were averaged together under "Average 8". Excluding MUSCLE, all methods were averaged across all datasets under "Average 10". The number of sequences are as follows: PDZ (14,950), blmb (17,200), p450 (21,013), adh (21,331), aat (25,100), rrm (27,610), Acetyltransf (46,285), sdr (50,157), zf-CCHH (88,345), rvp (93,681).

S5.4 Experiment 2b: Results on the 7 smallest CRW datasets

Table S6: **Average Alignment Error of UPP2 and Other MSA methods on Small to Medium RNA Datasets** The table shows the average alignment error across the seven small to medium RNA datasets.

	Avg (SPFN + SPFP)/2	Avg SPFN	Avg SPFP
UPP2-EarlyStop	0.101	0.101	0.101
UPP2	0.100	0.099	0.102
MAGUS	0.101	0.098	0.104
PASTA	0.117	0.108	0.126
MAFFT-linsi	0.115	0.111	0.119
MAFFT-ginsi	0.108	0.101	0.115
Clustal Omega	0.234	0.246	0.223
T-COFFEE	0.243	0.256	0.230
MUSCLE	0.193	0.199	0.187

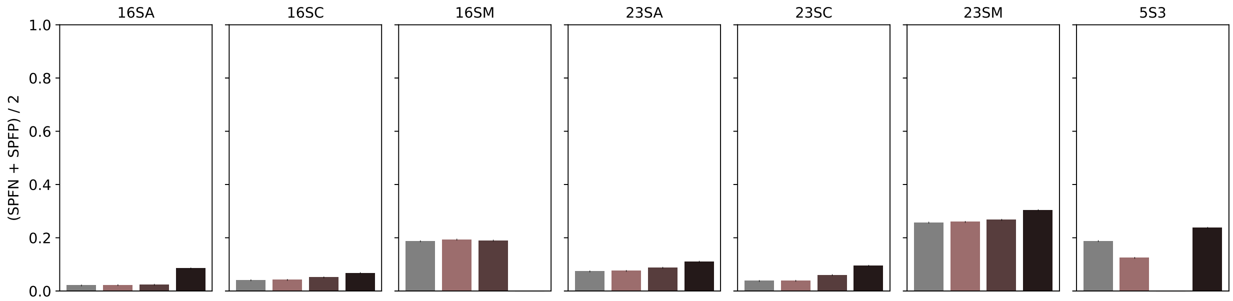

(a) Average alignment error

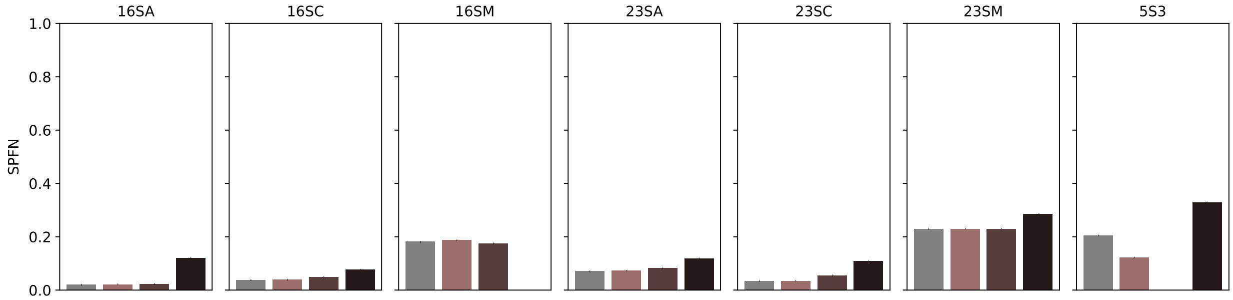

(b) SPFN alignment error

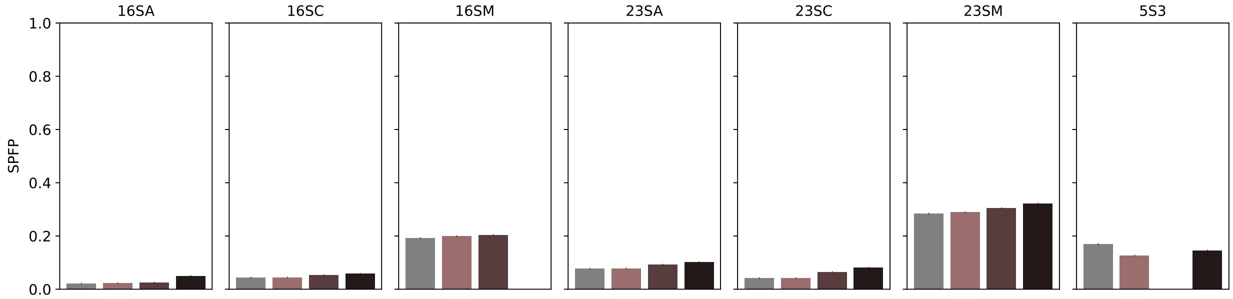

(c) SPFP alignment error

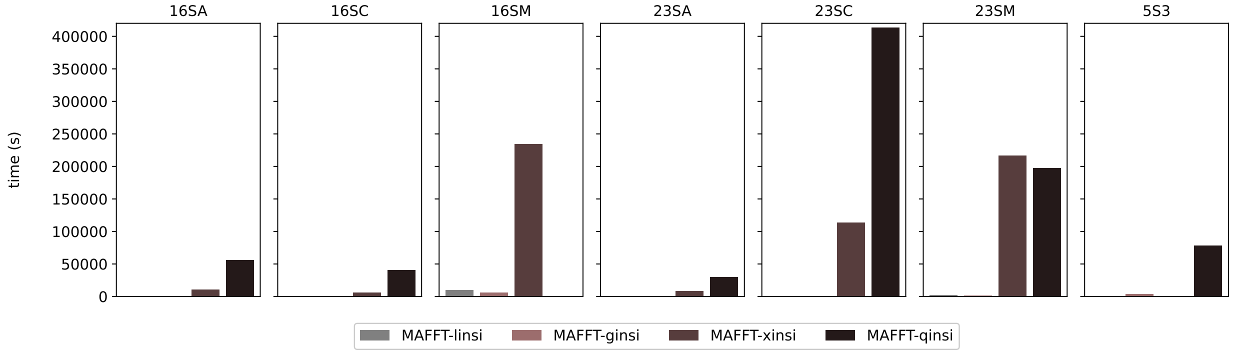

(d) Running time

Figure S28: **Alignment Error and Runtime of MAFFT Variants on Small to Medium RNA Datasets from the CRW Collection** These datasets are RNA datasets from the CRW collection with dataset sizes ranging from 214 to 5507. The individual dataset sizes are as follows: 16S.A(594), 16S.C(320), 16S.M(805), 23S.A(214), 23S.C(374), 23S.M(254), 5S.3(5507). Results not shown in 16S.M for MAFFT-qinsi and 5S.3 for MAFFT-xinsi are due to the methods not completing within the allotted time.

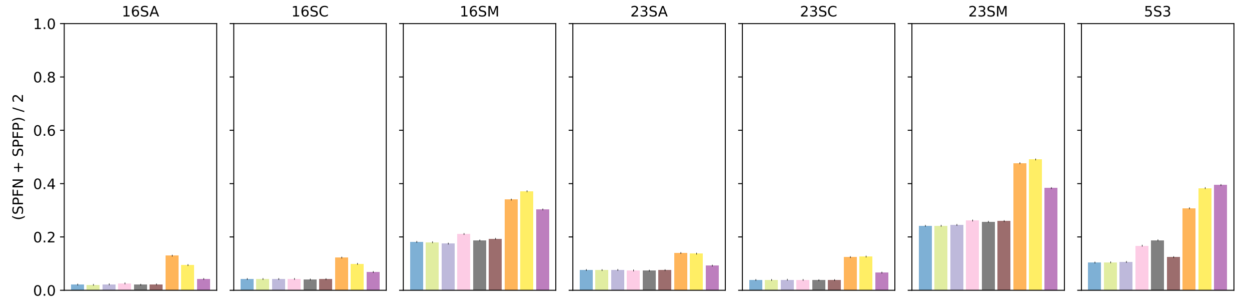

(a) Average alignment error

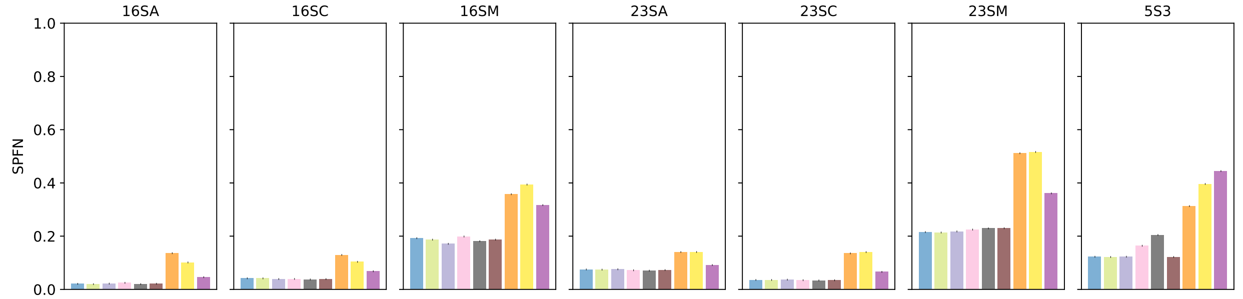

(b) SPFN alignment error

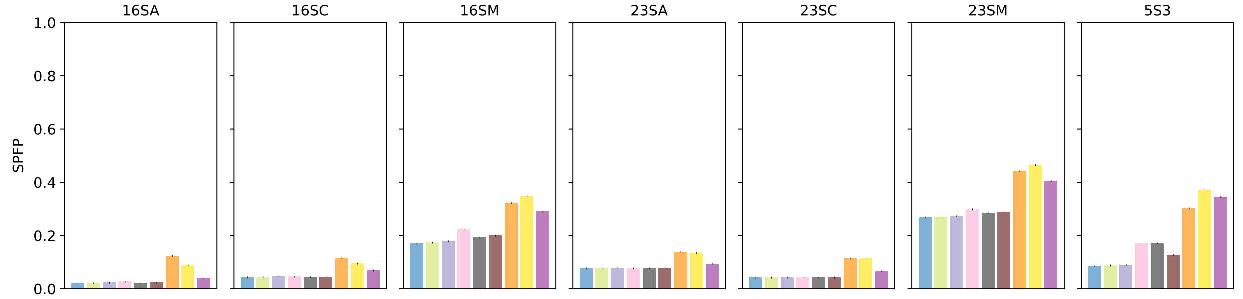

(c) SPFP alignment error

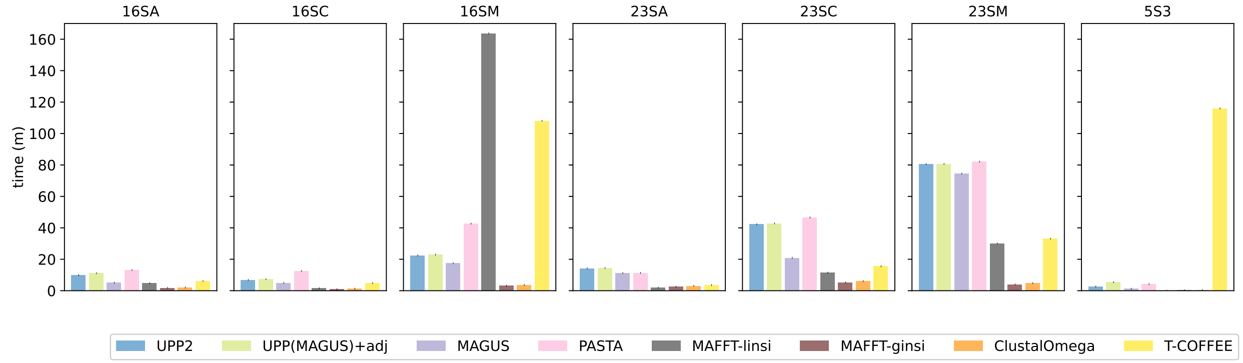

(d) Running time

Figure S29: **Alignment Error and Runtime of UPP2 Compared to Other MSA Methods on Small to Medium RNA datasets** These datasets are RNA datasets from the CRW collection with dataset sizes ranging from 214 to 5507. The individual dataset sizes are as follows: 16S.A(594), 16S.C(320), 16S.M(805), 23S.A(214), 23S.C(374), 23S.M(254), 5S.3(5507).

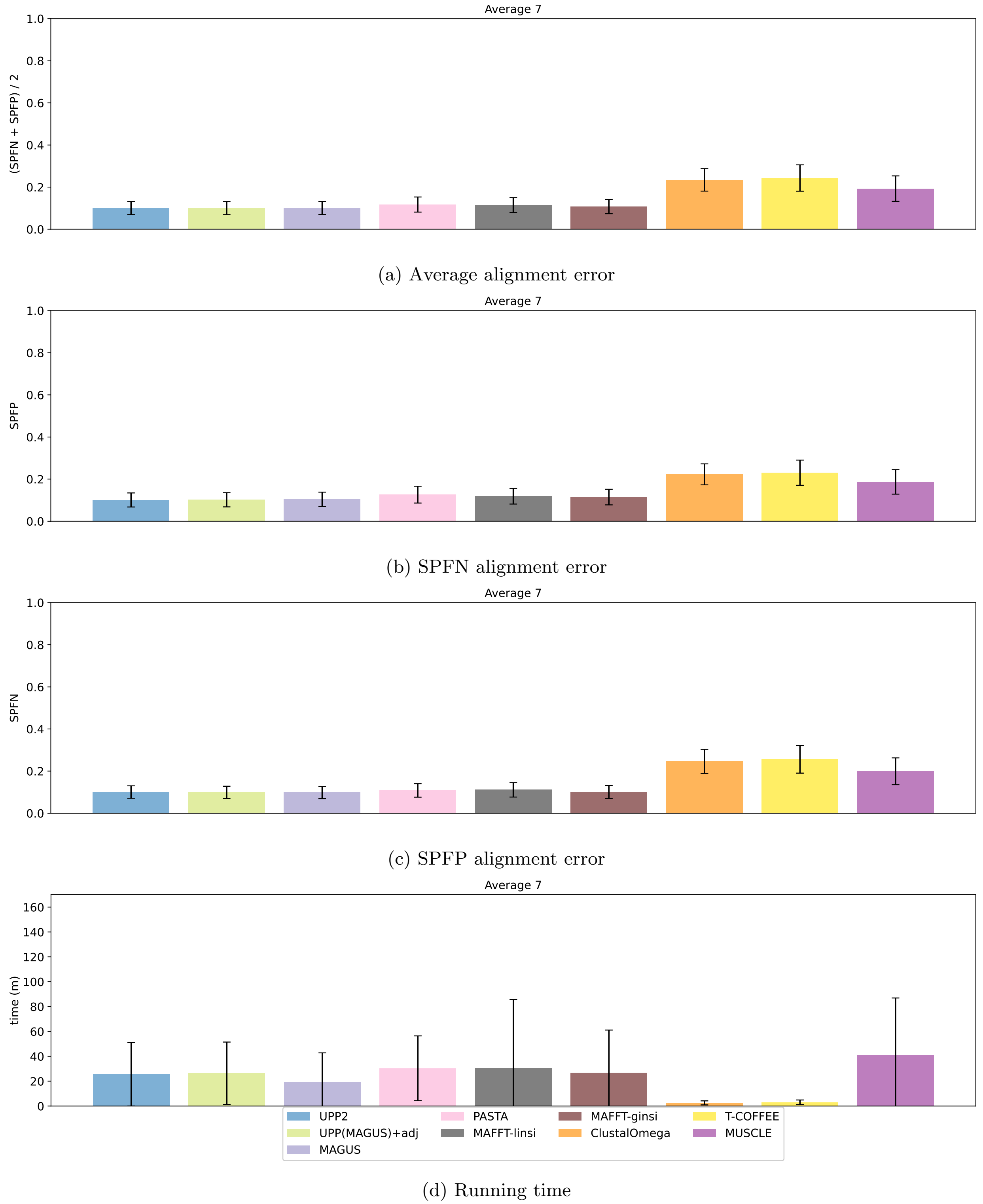

Figure S30: **Average Alignment Error and Runtime of UPP2 Compared to Other MSA Methods on Small to Medium RNA Datasets** These datasets are RNA datasets from the CRW collection with dataset sizes ranging from 214 to 5507. The individual dataset sizes are as follows: 16S.A(594), 16S.C(320), 16S.M(805), 23S.A(214), 23S.C(374), 23S.M(254), 5S.3(5507).

S5.5 Other experiments

Figure S31 compares methods on simulated datasets without any introduced fragmentation, showing both alignment error and running time; Figure S32 provides a closer look at the runtime comparison between UPP2 and UPP(MAGUS)+adj on these datasets. UPP2, UPP(MAGUS)+adj, MAGUS, and PASTA were the four leading methods across the simulated model conditions. Within this leading group of methods, MAGUS was the most accurate alignment method, closely followed by UPP2, PASTA, and UPP(MAGUS)+adj, in that order. Clustal Omega and T-COFFEE were the least accurate methods. MUSCLE, although more accurate than Clustal Omega and T-COFFEE, was less accurate than the leading group of four methods. UPP2 and MAGUS were the fastest methods, followed by Clustal-Omega and T-COFFEE. UPP(MAGUS)+adj was almost always the slowest, but PASTA, Muscle, and MAFFT linsi were typically also slow. Thus, if selecting from among the most accurate methods, UPP2 and MAGUS are the only ones that provide competitive accuracy and also fast running times.

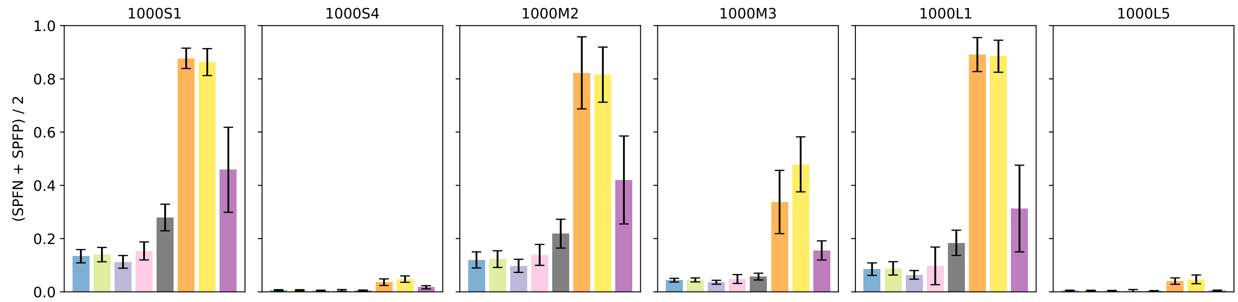

(a) Average alignment error

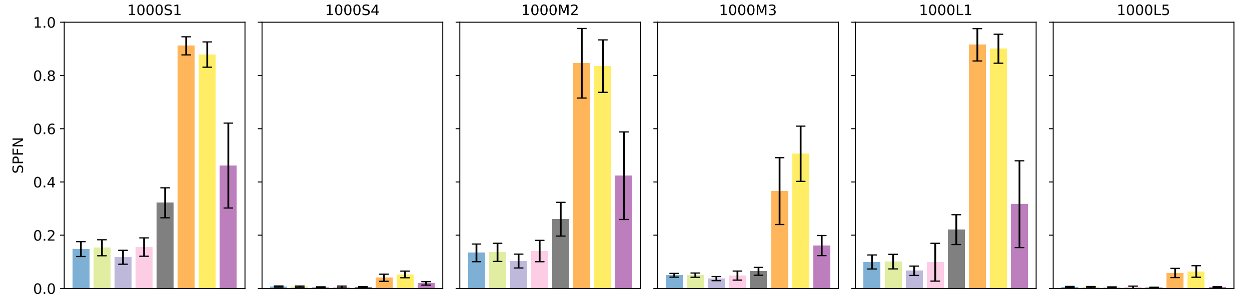

(b) SPFN alignment error

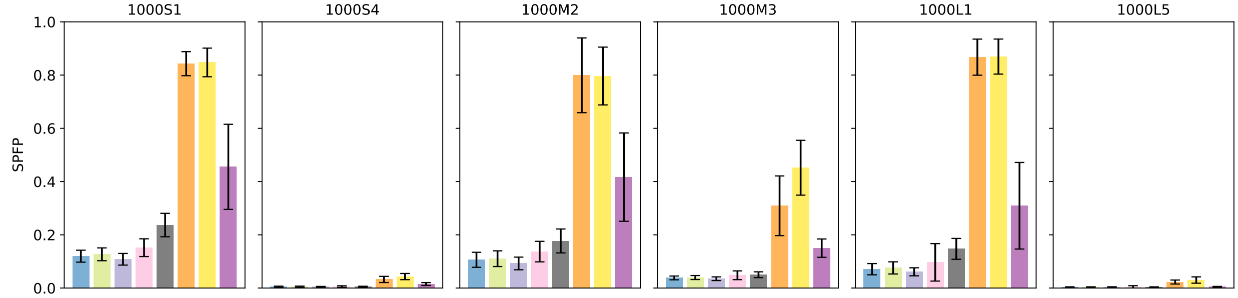

(c) SPFP alignment error

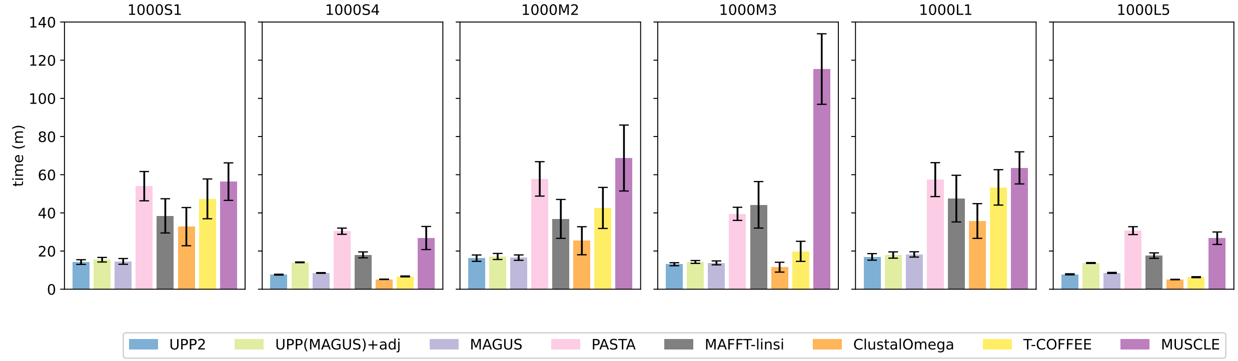

(d) running time

Figure S31: **Experiment 2a: UPP2 Compared to Other MSA Methods on Simulated Datasets without Fragmentation.** We show the alignment error and runtime of UPP2 (i.e., UPP(MAGUS)+adj+EarlyStop) compared to other alignment methods. All methods except T-COFFEE and MUSCLE were run in their default modes and with 16 threads, when possible. T-COFFEE was run using the regressive mode and MUSCLE was limited to 2 iterations. All datasets have 20 replicates each. The means are shown with error bars indicating standard error for alignment error and standard deviation for running time.

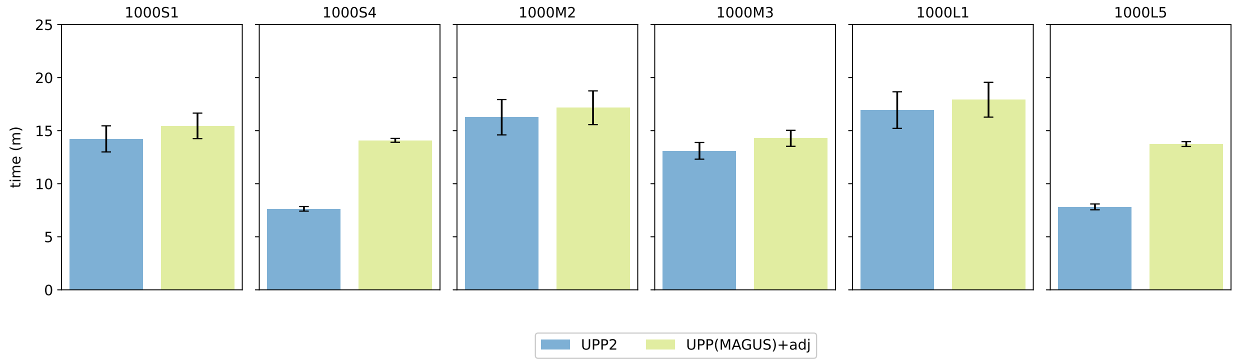

Figure S32: **Experiment 2a: Runtime of UPP2 and UPP(MAGUS)+adj on Simulated Datasets Without Fragmentation** UPP(MAGUS)+adj and UPP2 both use MAGUS backbone alignments, FastTree backbone trees, and adjusted bit-scores; they differ in their search strategies (EarlyStop vs all-against-all). All datasets have 20 replicates each. The means are shown with error bars indicating standard deviation for running time.

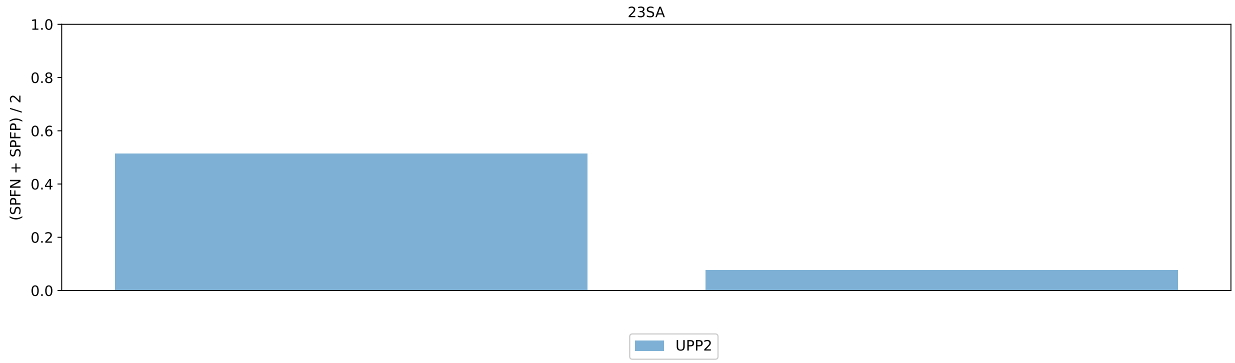

Figure S33: **Impact of Different Full-length Sampling Approaches** The RNA dataset 23S.A from the Comparative Ribosomal Website (CRW) is used as an example to illustrate the impact different backbone alignments can have on the alignment error of UPP2. As seen in Figure S7, the alignment is bimodal, with a peak for very short sequences and another peak with very long sequences. The standard approach (which works for many simulated datasets) of picking within 25% of the median length to define the backbone fails badly on this dataset. A modified approach of using 25% of the mode sequence length also fails, as is shown in this figure (left). In comparison, we show the current backbone selection strategy on the the right; see Section S7 for full details.

S6 Information about MSA failures

S6.1 RNA (CRW) Datasets

MAFFT variant failures On the largest 16S datasets (16S.3, 16S.T, and 16S.B.ALL), all runs of MAFFT-ginsi, MAFFT-xinsi, and MAFFT-qinsi failed. MAFFT-xinsi failed due to out of memory on 16S.3 and 16S.T, and failed due to an undiagnosable issue on 16S.B.ALL. MAFFT-qinsi timed out on 16S.3 after 7 days and ran out of memory on 16S.T and 16S.B.ALL. MAFFT-ginsi timed out after 7 days on all three of the large 16S datasets.

On the seven smaller datasets, only MAFFT-qinsi and MAFFT-xinsi had any failures. MAFFT-qinsi failed on 16S.M and MAFFT-xinsi failed on 5S.3, each due to reaching the 7-day running time limit.

S6.2 Homfam Datasets

Of the ten Homfam datasets, T-COFFEE failed to run on eight of them due to memory errors. MUSCLE failed to run on the two largest Homfam datasets due to memory errors. We describe the exact errors we encountered below with additional information about the system they ran on.

MUSCLE failures MUSCLE had trouble running on the two largest Homfam datasets (zf-CCHH and rvp) with signal 11, also known as segmentation fault.

T-COFFEE failures All T-COFFEE runs were done using *T-COFFEE Version_13.45.0.4846264 (2020-10-15 17:52:11 - Revision 5becd5d - Build 620) – regressive mode* in its default mode. They were run on the Illinois Campus Cluster using Singularity. The particular node that the runs were done on had 16 cores available with 128 GB of RAM on a Linux kernel 3.10.0-1160.42.2.el7.x86_64 version #1 SMP Tue Aug 31 20:15:00 UTC 2021. The Singularity image is located at `https://github.com/MinhyukPark/Containers/blob/master/bio.bootstrap`. We ran T-COFFEE after removing the cache directory at $HOME and specifying -cache=no as instructed by the T-COFFEE website, but we were unable to get an output alignment. We also ran T-COFFEE on different nodes on the Campus Cluster, as well as without the Singularity image and installing T-COFFEE, Clustal Omega, and MAFFT locally, but all analyses resulted in the same error.

We also note that on the same system, not even the ROSE datasets could run, resulting in a "free(): double free detected in tcache 2" error. We noticed that by removing sequences from the initial set of sequences, at some point the alignment becomes small enough where T-COFFEE does run without error. These errors and findings were observed with both DNA and Protein sequences.

Specific T-COFFEE outputs by Homfam dataset On the PDZ dataset, T-COFFEE fails with "!All Jobs collected" in its output without any indication as to why T-COFFEE was unable to produce any output alignment. The only message indicative of error is "mv: cannot create regular file '0': File exists" in several places in its output.

On the blmb, adh, and Acetyltransf datasets, T-COFFEE fails with "double free or corruption (!prev)" error, which is a typical memory error. It is able to compute the guide tree and weights before segfaulting on computing the MSA step.

On the p450, aat, sdr, zf-CCHH, and rvp datasets, T-COFFEE fails with "double frcee or corruption (!prev)" error during the compute guide tree step.

On the rrm dataset, T-COFFEE fails with "–ERROR: : Impossible to run dynamic.pl" during the compute MSA step.

S7 Backbone Query Split Algorithm Details

The sequence length histograms of biological datasets can deviate substantially from the distributions seen in simulated datasets. As a result, standard approaches for selecting the "full-length" sequences to put in the backbone can fail to produce good results. Figure S7 shows the histogram for the RNA dataset 23S.A from the Comparative Ribosomal Website [1], and is an example of this phenomenon. A new sampling strategy is described here that produces a more reliable backbone selection, and a comparison between using this new strategy compared to older strategy is given in Figure S33. Here we describe the new strategy.

We used a sliding window procedure, as follows. For every sequence s, we count the number of sequences that have lengths at least 75% and at most 125% of the length of s. Once we have noted the counts for each sequence in our dataset, we select whichever sequence had the highest count as our representative full-length sequence. Then, any sequence in our dataset that is at least 75% as long as our representative full-length sequence is considered full-length. Below we show the pseudocode for finding the representative full-length sequence given a dataset. After this procedure is run, any sequence in the dataset that is at least 75% is categorized as full-length and the remaining sequences as fragmentary.

Algorithm 1 The pseudocode for selecting the representative full-length sequence from a given dataset

```
S: Array of sequences in our dataset from the first to the Lth sequence
function FindRepresentativeSequence(S[1..L])
    max_count ← 0                      ▷ This keeps track of the size of the largest window
    max_index ← −1                     ▷ This keeps track of the index of the sequence with the largest window
    for s ∈ S do
        min_bound = s.length ·0.75     ▷ This is the minimum bound for the current window
        max_bound = s.length ·1.25     ▷ This is the maximum bound for the current window
        current_count ← 0              ▷ This keeps track of the size of the current window
        for s′ ∈ S do
            if s'.length ≥ min_bound  and  s'.length ≤ max_bound then    ▷ if s' in window
                current_count ← current_count + 1
            end if
        end for
        if current_count ≥ max_count then    ▷ If current window is the larger
            max_count ← current_count
            max_index ← s.index
        end if
    end for
    return S[max_index]                ▷ This is the representative full-length sequence
end function
```

References

[1] Jamie J Cannone, Sankar Subramanian, Murray N Schnare, James R Collett, Lisa M D'Souza, Yushi Du, Brian Feng, Nan Lin, Lakshmi V Madabusi, Kirsten M Müller, Nupur Pande, Zhidi Shang, Nan Yu, and Robin R Gutell. The Comparative RNA Web (CRW) Site: an online database of comparative sequence and structure information for ribosomal, intron, and other RNAs. *BMC Bioinformatics*, 3(1):1–31, 2002.

[2] E. Garriga et al. Zenodo repository for the T-COFFEE Regressive paper, 2018. `https://zenodo.org/record/3271452#.YrYD-RPMLmo`, last accessed June 24, 2022.

[3] Chengze Shen, Minhyuk Park, and Tandy Warnow. WITCH: improved multiple sequence alignment through weighted consensus HMM alignment. *Journal of Computational Biology*, 2022. https://doi.org/10.1089/cmb.2021.0585.
